# Archaeal and bacterial communities in deep-sea hydrogenetic ferromanganese crusts on old seamounts of the northwestern Pacific

**DOI:** 10.1371/journal.pone.0173071

**Published:** 2017-02-24

**Authors:** Shota Nitahara, Shingo Kato, Akira Usui, Tetsuro Urabe, Katsuhiko Suzuki, Akihiko Yamagishi

**Affiliations:** 1 Department of Molecular Biology, Tokyo University of Pharmacy and Life Science, Hachioji, Tokyo, Japan; 2 Ore Genesis Research Unit, Project Team for Development of New-generation Research Protocol for Submarine Resources, Japan Agency for Marine-Earth Science and Technology (JAMSTEC), Yokosuka, Kanagawa, Japan; 3 Center for Advanced Marine Core Research, Kochi University, Kochi-shi, Kochi, Japan; 4 Department of Earth and Planetary Science, The University of Tokyo, Hongo, Bunkyo-ku, Tokyo, Japan; Wageningen University, NETHERLANDS

## Abstract

Deep-sea ferromanganese crusts are found ubiquitously on the surface of seamounts of the world’s oceans. Considering the wide distribution of the crusts, archaeal and bacterial communities on these crusts potentially play a significant role in biogeochemical cycling between oceans and seamounts; however little is known about phylogenetic diversity, abundance and function of the crust communities. To this end, we collected the crusts from the northwest Pacific basin and the Philippine Sea. We performed comprehensive analysis of the archaeal and bacterial communities of the collected crust samples by culture-independent molecular techniques. The distance between the sampling points was up to approximately 2,000 km. Surrounding sediments and bottom seawater were also collected as references near the sampling points of the crusts, and analyzed together. 16S rRNA gene analyses showed that the community structure of the crusts was significantly different from that of the seawater. Several members related to ammonia-oxidizers of *Thaumarchaeota* and *Betaproteobacteria* were detected in the crusts at most of all regions and depths by analyses of 16S rRNA and *amoA* genes, suggesting that the ammonia-oxidizing members are commonly present in the crusts. Although members related to the ammonia-oxidizers were also detected in the seawater, they differed from those in the crusts phylogenetically. In addition, members of uncultured groups of *Alpha*-, *Delta*- and *Gammaproteobacteria* were commonly detected in the crusts but not in the seawater. Comparison with previous studies of ferromanganese crusts and nodules suggests that the common members determined in the present study are widely distributed in the crusts and nodules on the vast seafloor. They may be key microbes for sustaining microbial ecosystems there.

## Introduction

Deep-sea ferromanganese crusts, which are iron and manganese oxyhydroxide coatings, are found ubiquitously on the world’s seamounts between 1000–4000 m water depth [1]. Hydrogenetic ferromanganese crusts grow very slowly (1–10 mm/Myr (millions of years)) as they are chemical precipitates from the overlying seawater in an oxic environment [1]. In fact, thick crusts (>10 cm) are commonly found only on old rocks of several tens or over a hundred Myr [2], while thin crusts (*c*. 10 μm) are found on the surfaces of freshly made rocks near oceanic ridges where new plate material is being created [3]. In contrast, diagenetic manganese deposits are generated by diagenetic processes in the underlying sediments. Hydrothermal manganese deposits are precipitated from hydrothermal fluids at and around deep-sea vent fields. These diagenetic and hydrothermal deposits grow tens to hundreds times faster than the hydrogenetic deposits [1, 4]. Ball-shaped manganese nodules are found on deep-sea sediments as hydrogenetic and/or diagenetic deposits [1, 4]. Major chemical compositions of the hydrogenetic ferromanganese crusts and nodules (Fe, Mn and Co rich), hydrothermal manganese or iron deposits (Si and Fe or Mn rich), and surrounding dee-sea sediments (Si and Ca rich) are different [1, 4]. The hydrogenetic ferromanganese crusts and nodules (hereafter, crusts and nodules) contain significant quantities of economically valuable elements, such as Co, Ni, Pt, and rare earth elements, besides Fe and Mn. Therefore, they are of great interest as mineral resources (e.g. [5]). The crusts are of varied thickness, and coat not only on basement basaltic rocks but also limestone and other substrates [1, 4]. The crusts cover considerable areas of the global seafloor [1, 4].

Metabolic functions of the members in microbial communities of the crusts are unclear because only 16S rRNA gene data is available for them, and no functional gene analyses (such as *amoA*) have been reported to date. In addition, it is still largely unknown what species of bacteria and archaea are commonly present among the crust microbial communities or between the crust and nodule communities in various areas. In general, a small number of microbiological studies have been undertaken on the crusts probably due to technical difficulties to collect samples. Recent reports of 16S rRNA gene analyses have shown that a large part of membership of the crust communities are different from those in the surrounding sediments and bottom seawater [6, 7]. The phylogenetic diversity of the crust communities is much higher than that of the seawater communities [6, 7]. Highly diverse microbes in a basaltic rock covered with thin Mn oxides have also been reported [8]. Nitahara et al. [6] have suggested that ammonia oxidizers sustain the crust microbial communities as primary producers. Nitahara et al. [6] have also reported that 16S rRNA genes related to ammonia oxidizers are also present in the bottom seawater as primary producers; however, these genes in the seawater are phylogenetically different from those detected in the crusts at a species level at least [6]. This imply that physiological characteristics, such as life styles (i.e., free-living or surface-attached) and nutrient requirements, of the ammonia oxidizers in the crusts differ from those in the seawater. A comparative analysis has shown that the microbial community of the crust is different from those of hydrothermal deposits such as iron-rich mats and sulfide chimneys probably due to difference in temperature and energy sources [9]. 16S rRNA gene analyses of the nodules have been performed [10–13]. These studies showed the presence of highly diverse microbes and putative ammonia oxidizers in the nodules.

The northwestern Pacific seafloor near Japan was chosen as the study area because of the existence of two areas of deep seafloor of different ages and tectonic settings, with elevated, old seamounts. We hypothesized that there are common members among the crusts under the similar environmental conditions, such as low temperature and the presence of ferromanganese oxides. To test this hypothesis, we collected eight samples of the crusts at three seamounts, and performed comparative analysis of microbial communities by culture-independent molecular techniques targeting to 16S rRNA and *amoA* genes. By comparing crusts collected and analyzed using the same procedures we were able to look for general trends, since methodological biases had been minimized. In addition, surrounding sediments and bottom seawater were collected as references and analyzed together with the crust samples. The presence of ammonia oxidizers in a crust have been suggested previously [6]. To test if the ammonia oxidizers are commonly present on the crusts, we performed *amoA* gene analyses. The present study reports, for the first time, the abundance and phylogenetic diversity of ammonia oxidizers in the crust microbial communities based on *amoA* gene analyses.

## Materials and methods

### Sampling

Samples of the crusts, surrounding oligotrophic sediments, and bottom seawater (Table 1) were collected from three study areas, i.e. the Takuyo-Daigo Seamount, the Ryusei Seamount, and the Daito Ridge during cruises NT09-02 with the research vessel (R/V) *Natsushima* (JAMSTEC) in February 2009, KY11-02 with the R/V *Kaiyo* (JAMSTEC) in February 2011, and NT12-25 with the R/V *Natsushima* in October 2012. During the cruises, the remotely operated vehicle (ROV) *Hyper-Dolphin* (JASMTEC) collected the samples and, using a CTD-DO sensor, measured temperature, salinity, and dissolved oxygen (DO) concentration during sampling dives. The locations for sample collection were within the exclusive economic zone of Japan. No specific permits were required for the described field studies and sample collection. The field studies did not involve endangered or protected species.

**Table 1:** List of the samples used in the present study.

Sample ID	Depth (m)	Latitude (N)	Longitude (E)	Sampling date (m/d/y)	Sampling site	Sample type	Sample number
MnTk12	1200 m	22°45.742'	153°16.940'	2/17/2009	Takuyo-Daigo Smt.	Ferromanganese crust	HPD#957-R16
MnTk14	1419 m	22°44.656'	153°16.113'	2/17/2009	Takuyo-Daigo Smt.	Ferromanganese crust	HPD#957-R05
MnTk22	2209 m	22°42.504'	153°15.381'	2/15/2009	Takuyo-Daigo Smt.	Ferromanganese crust	HPD#955-R04
MnTk30	2991 m	22°40.984'	153°14.617'	2/11/2009	Takuyo-Daigo Smt.	Ferromanganese crust	HPD#953-R01
MnRy12	1194 m	25°32.550'	135°34.687'	2/4/2011	Ryusei Smt.	Ferromanganese crust	HPD#1244-R08
MnRy21	2079 m	25°31.745'	135°33.570'	2/3/2011	Ryusei Smt.	Ferromanganese crust	HPD#1243-R02
MnDi15	1461 m	25°40.604'	133°15.238'	10/8/2012	Daito Ridge	Ferromanganese crust	HPD#1444-R06
MnDi18	1838 m	25°40.078'	133°15.378'	10/3/2012	Daito Ridge	Ferromanganese crust	HPD#1442-R01
SdTk14	1434 m	22°44.610'	153°15.996'	2/17/2009	Takuyo-Daigo Smt.	Sediment	HPD#957-C01
SdTk30	2991 m	22°40.985'	153°14.618'	2/11/2009	Takuyo-Daigo Smt.	Sediment	HPD#953-C01
SdRy15	1525 m	25°32.558'	135°34.687'	2/4/2011	Ryusei Smt.	Sediment	HPD#1244-C01
SdRy21	2081 m	25°31.734'	135°33.570'	2/3/2011	Ryusei Smt.	Sediment	HPD#1243-C01
SdDi14	1409 m	25°40.771'	133°15.210'	10/8/2012	Daito Ridge	Sediment	HPD#1444–01
SdDi18	1832 m	25°40.086'	133°15.371'	10/3/2012	Daito Ridge	Sediment	HPD#1442-C01
AsTk14	1434 m	22°44.611'	153°15.997'	2/17/2009	Takuyo-Daigo Smt.	Seawater	HPD#957-W01
AsTk30	2991 m	22°40.986'	153°14.619'	2/11/2009	Takuyo-Daigo Smt.	Seawater	HPD#953-W01
AsRy16	1553 m	25°32.558'	135°34.687'	2/4/2011	Ryusei Smt.	Seawater	HPD#1244-W01
AsRy21	2079 m	25°31.745'	135°33.570'	2/3/2011	Ryusei Smt.	Seawater	HPD#1243-W01
AsDi16	1638 m	25°40.368'	133°15.269'	10/8/2012	Daito Ridge	Seawater	HPD#1444-W01
AsDi18	1838 m	25°40.078'	133°15.378'	10/3/2012	Daito Ridge	Seawater	HPD#1442-W01

The Takuyo-Daigo Seamount is a flat-topped seamount (guyot) located in the northwestern Pacific. This area is one of the oldest seafloors of the world (>150 Myr old) [14]. The age of the seamount itself is approximately 100 Myr, as determined by Ar-Ar dating of the seamount’s basalt [15]. Osmium-isotope dating has indicated that the crust is still growing [15]. The Ryusei Seamount and the Daito Ridge are located at the Philippine Sea which is much younger than Pacific basin (>80 Myr old [14]). Concentrations of major components such as Mn and Fe of the crust samples are typical of hydrogenetic crusts [16]. The samples were collected at the Takuyo-Daigo Seamount at water depths between 1000 m and 3000 m, at the Ryusei Seamount at water depths between 1200 m and 2100 m, and at the Daito Ridge at water depths between 1400 m and 1800 m. It should be noted that the 16S rRNA gene data of microbial communities of the Takuyo-Daigo Seamount at 3000 m have been already reported [6]; they were re-analyzed here together with new data from this study.

As previously described [6], the crust samples were collected using a manipulator of the ROV, while around those sampling points sediment samples were collected using a push-core sediment sampler, and seawater samples were collected using NISKIN samplers at 1–2 m above the seafloor. The surface of the crust samples was washed three times with seawater filtered with a 0.2 μm-pore polycarbonate membrane to remove loosely attached particles, potentially containing contaminants derived from sediments and seawater. The 0–<5 mm surface parts of the crusts were peeled off using a sterile hammer and chisel. These surface parts were stored in sterile DNA/RNA-free plastic tubes in a bio-clean booth onboard the vessel. The cores of the surrounding sediments were subsampled (0–50 mm from the top) using a sterile spatula and those subsamples put into sterile DNA/RNA-free plastic tubes. Each 2 L sample of ambient seawater was filtered with 0.2 μm-pore polycarbonate membranes. The filters were then cut into quarters, and put into sterile DNA/RNA-free plastic tubes. Tubes containing the crusts, sediments and filters were stored at -80°C until DNA extraction.

### DNA extraction and clone library construction

DNA extraction and PCR clone library construction were performed as described previously [6]. In brief, genomic DNA was extracted from the samples of the crusts, sediments, and membrane filters using a FastDNA spin kit for soil (MP Biomedicals, Santa Ana, CA). Partial 16S rRNA genes were amplified by PCR using the universal primer set, Uni515F and Uni1406R, for bacteria and archaea. In addition to amplifying the 16S rRNA genes, we performed *amoA* gene analysis. Partial archaeal and bacterial *amoA* genes were amplified by PCR using the following primer sets: amoA1Fmod and amoArNEW for bacterial *amoA* genes, and Arch-amoAF and Arch-amoAR for archaeal *amoA* genes, as described previously [17, 18]. The PCR products were cloned using a TOPO TA cloning kit (Invitrogen, Carlsbad, CA), and randomly selected clones were sequenced by Sanger sequencing as described previously [6].

### Sequence analysis

For the 16S rRNA gene sequences, QIIME ver. 1.9 [19] was used for operational taxonomic unit (OTU) clustering at a 97% similarity level (i.e. a definition of species level) using the UCLUST algorithm [20] with default parameters. Taxonomic affiliation of the OTUs were performed using the Greengenes database [21] equipped with QIIME. Comparative community analysis by principal coordinate analysis (PCoA) and cluster analysis were also performed by QIIME using the weighted UniFrac method [22]. Alignments of the OTUs for PCoA and cluster analysis were generated using PyNAST [23] equipped with QIIME. The smallest number of the sequences among the samples was used as the rarefied sequence number for PCoA. Potential chimeric sequences were identified using QIIME via ChimeraSlayer [24], and were removed in all analyses. Statistical analysis for assessment of the differences between the two libraries was performed by using the P (phylogenetic) test [25] and UniFrac significance test [22]. ANOSIM (Analysis of similarities) test [26] with 9999 permutations was performed for assessment of differences between two groups of libraries. To assess the difference between diversity values, t-tests were performed using R software (https://www.R-project.org).

The *amoA* sequences were aligned by MUSCLE ver. 3.8.31 [27], and the alignment was then used for OTU clustering at a 97% similarity level by mothur ver. 1.36.1 [28]. Potential chimeric sequences of *amoA* genes were detected using mother via UCHIME [29] and removed.

For 16S rRNA and *amoA* genes, Good’s coverage, Chao1 species richness estimates, Shannon diversity index, and shared numbers of OTUs were calculated using mothur. Sequences closely related to our detected sequences were collected from public databases, the public and our sequences were then aligned using MUSCLE. Sequences of the cultured species that were most closely related to the detected sequences are also included in the alignment. Gap positions were removed from the alignment using trimAl ver. 1.2 [30]. Phylogenetic trees were constructed using FastTree ver. 2.1.8 [31] with a GTR nucleotide substitution model.

### qPCR

Copy numbers of 16S rRNA genes were determined using quantitative PCR (qPCR) with the following primers and TaqMan probe set: Bac1369F-TM1389F-Prok1492R for bacterial 16S rRNA genes [32] and Arc349F-Arc516F-Arc806R for archaeal 16S rRNA genes [33], as described previously [6]. Regression coefficient (*r*^2^) values of the standard curves for bacterial and archaeal 16S rRNA genes were 0.995 and 0.999, respectively. Copy numbers of archaeal and bacterial *amoA* genes were also determined using qPCR with the following primers: amoA1Fmod and amoArNEW for bacterial *amoA* genes, and Arch-amoAF and Arch-amoAR for archaeal *amoA* genes, as described previously [17, 18]. A detected clone of bacterial (MnTk30B_44) or archaeal *amoA* gene (MnTk30A_04) was used for standard curve construction (*r*^2^ values for bacterial and archaeal *amoA* genes were 0.997 and 0.989, respectively).

Total numbers of bacterial and archaeal cells were estimated from the 16S rRNA gene qPCR results, based on the median of 4 copies/genome for bacteria and 1 copy/genome for archaea, as reported in a curated database of ribosomal RNA operons (rrnDB) version 4.4.4 [34] as 1 genome/cell. Numbers of cells of bacterial and archaeal ammonia oxidizers were also estimated from the qPCR results, based on 2 copies/genome for bacterial ammonia oxidizers and 1 copy/genome for archaeal ammonia oxidizers, as reported in five complete genomes of ammonia-oxidizing bacteria in *Betaproteobacteria* (NC_004757, *Nitrosomonas europaea*; NC_008344, *Nitrosomonas eutropha*; NC_015222, *Nitrosomonas* sp. AL212; NC_015731, *Nitrosomonas* sp. Is79A3; NC_007614, *Nitrosospira multiformis*), and six complete genomes of ammonia-oxidizing archaea in *Nitrosopumilales* (CP003843, Candidatus *Nitrosopumilus* sp. AR2; CP000866, *Nitrosopumilus maritimus*; CP007026, Candidatus *Nitrosopelagicus brevis* CN25; CP003842, Candidatus *Nitrosopumilus koreensis* AR1; CP011070, Candidatus *Nitrosopumilus* sp. NF5).

### Accession numbers

The nucleotide sequences of 16S rRNA and *amoA* genes determined in the present study have been deposited in the DDBJ database under the following accession numbers: LC138362– LC139974 for 16S rRNA gene clones and LC139975–LC140541 for *amoA* gene clones.

## Results

### Sample description

Crusts were commonly observed on the slope of the three regions, i.e. the Takuyo-Daigo Seamount, the Ryusei Seamount, and the Daito Ridge at every depth of the dives. Collected crusts were all >1 cm thick (S1 Fig). The basement rocks of the crusts were basalts or limestones. The crusts were round or slab-shaped.

### Physicochemical properties of the water column

Water depth profiles of temperature, salinity and pH were similar for the three regions (S2 Fig). Temperature decreased from 4°C at a depth of 1000 m to 1–2°C at greater depth. Salinity was least (<34.2) at 600–800 m deep, and then increased gradually as depth increased (>34.6). An oxygen minimum zone (OMZ; 27%–29% of the DO concentrations of the surface seawater) was observed at 900–1100 m deep, and then DO concentration increased gradually with increasing depth. The observed depth profiles of temperature, salinity, and DO concentration were consistent with trends documented for pelagic areas in the Pacific [35].

### Quantitative PCR

To estimate total numbers of archaeal and bacterial cells, and those of ammonia-oxidizing bacteria and archaea (AOB and AOA, respectively), qPCR targeting 16S rRNA and *amoA* genes was performed. The qPCR results are summarized in S1 Table. In brief, the total number of archaeal and bacterial cells was estimated to be 10^7^ to 10^8^ cells/g for the crust samples, 10^8^ to 10^9^ cells/g for the sediment samples, and 10^2^ to 10^3^ cells/ml for the bottom seawater samples. Of the total number of archaeal and bacterial cells, archaea made up 8 to 89% in the crusts, 28 to 91% in the sediments, and 3 to 60% in the seawater. The total number of ammonia oxidizers (i.e. sum of AOB and AOA) was estimated to be up to 10^7^ cells/g for the crusts and sediments, and 10^2^ cells/ml for the seawater. Of all ammonia oxidizers, AOA made up 24 to 72% in the crusts, 7 to 14% in the sediments, and 100% in the seawater (bacterial *amoA* was not detected). Of the total number of archaeal and bacterial cells, ammonia oxidizers made up 1.6 to 7.3% in the crusts, 0.4 to 2.9% in the sediments, and 4.7 to 7.1% in the seawater.

### 16S rRNA gene clone libraries

To determine the phylogenetic diversity and community structures of the collected samples, we performed clone library analyses targeting 16S rRNA genes. Results of the PCR clone library analyses are summarized in S2 Table. A list of the 16S rRNA gene clones corresponding to each OTU is shown in S3 Table. Phylogenetic trees for all OTUs are shown in S3 Fig.

#### Alpha diversity metrics

Phylogenetic diversity of the seawater libraries was lower than that of the crust and sediment libraries as shown by Chao1 species richness estimates, the Shannon diversity index, and rarefaction curves, with the exception of a few libraries (S4 Fig, S2 Table). The alpha diversity of the crust libraries was comparable to that of the sediment libraries. The Chao1 and Shannon values for the crust, sediment and seawater samples were not correlated with depth (*r*^2^, <0.4). Greater numbers of samples at each depth are needed to assess the correlation between gene copy number and water depth.

Coverage values were low in the libraries of crusts (45 to 63%) and sediments (45.5±11.9%), and not particularly high even in the seawater libraries (31 to 65%) (S2 Table), indicating that the detected clones represented only the relatively abundant microbes in the communities. This is consistent with the unsaturated rarefaction curves (S4 Fig). In other words, there are considerable numbers of unrecovered microbes that make up minor components in the communities, especially for those of the crusts and sediments.

#### Difference in community structures among habitat types

**Fig 1:**
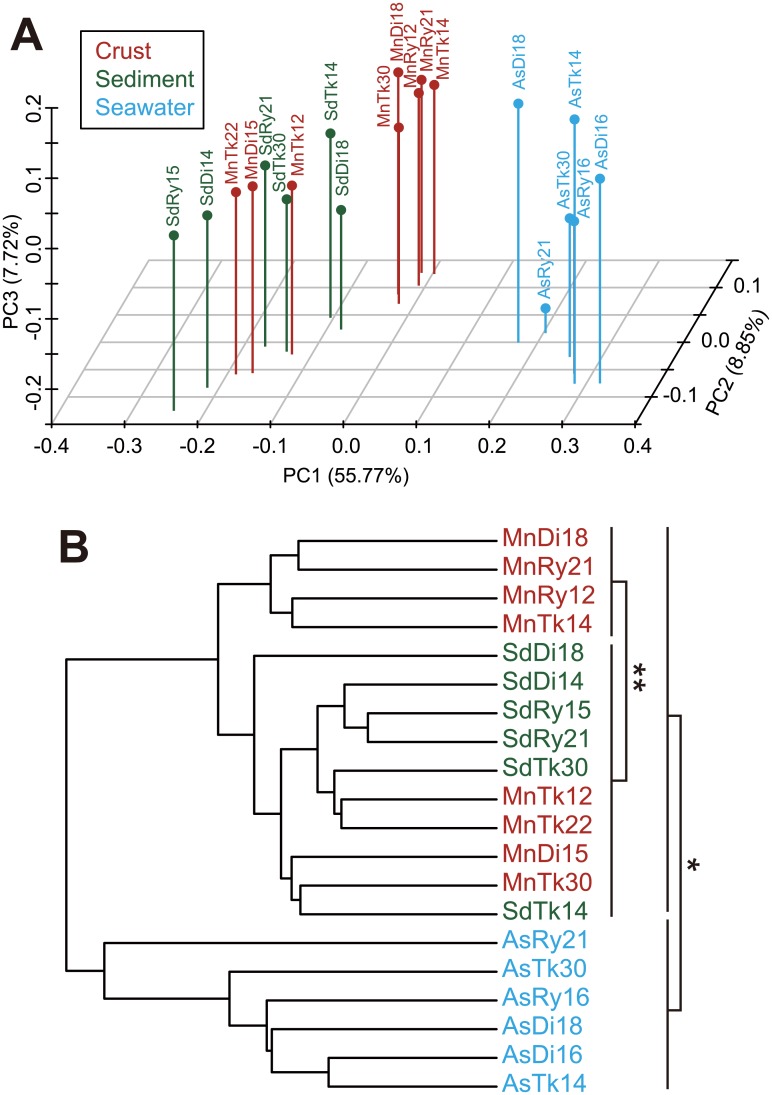
Comparison of community structure using the weighted UniFrac method based on the 16S rRNA gene clone library data. (A) 3D plot resulting from PCoA. The percentages in the axis labels represent the percentages of variation explained by the first, second, and third principal coordinates (PC1 to PC3, respectively). (B) UPGMA (unweighted pair group method using average linkages) tree resulted from cluster analysis. * and ** indicate *p* values of <0.0001 and <0.005, respectively, determined by ANOSIM test between two groups of libraries. Data for the crusts (red), sediments (green), and seawater (blue) are shown. The rarefied number of sequences used in the calculation was 51 (i.e., the minimum number of clones for each clone library).

**Fig 2:**
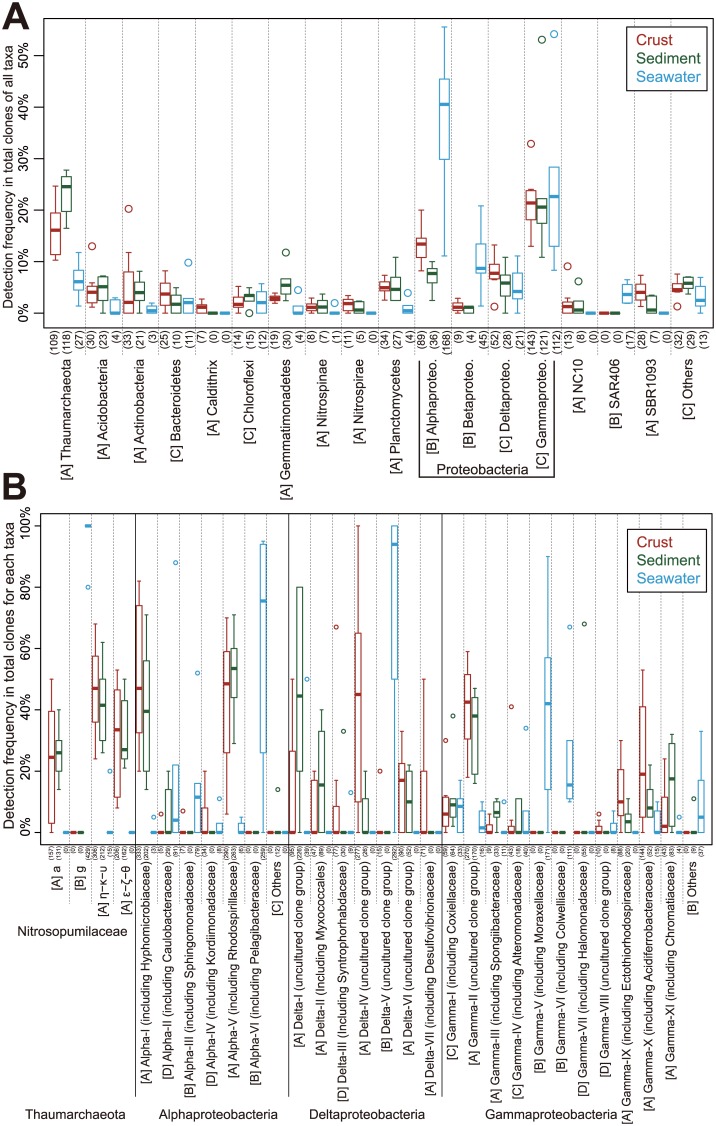
Detection frequency of clones for each taxonomic group in the libraries of the sample types. Data for the crusts (eight libraries combined; red), sediments (six libraries combined; green), and seawater (six libraries combined; blue) are shown. Numbers in parentheses indicate the total number of clones for each category. (A) Percentages of the clones affiliated with each phylum or each class of *Proteobacteria* in the total number of analyzed clones are shown in box plots. (B) Percentages of the clones affiliated with each group in the total number of the clones affiliated in each taxon are shown in box plots. The groupings for *Nitrosopumulaceae*, *Alpha*-, *Delta*-, and *Gammaproteobacteria* are based on the phylogenetic trees (S3A–S3C and S3I Fig). The detected taxa were summarized into the four categories: [A] the taxa whose relative abundance in the crusts and/or sediments is higher than that in the seawater, [B] those whose relative abundance in the seawater is higher than that in the crusts and sediments, [C] the taxa whose relative abundance is similar among the three types of the samples, and [D] the others.

Community structures of the crust and sediment samples were significantly different from those of the seawater samples as shown by PCoA and cluster analysis using the weighted UniFrac method (Fig 1, S4 Table). This difference in the community structures could reflect the presence/absence of each clone (S3 Fig), and each clone’s relative abundance (Fig 2). To address what taxa cause the observed difference in the community structures, we summarized the detected taxa into four categories (Fig 2); (A) the taxa whose relative abundance in the crusts and/or sediments is higher than that in the seawater, (B) those whose relative abundance in the seawater is higher than that in the crusts and sediments, (C) the taxa whose relative abundance is similar among the three types of the samples, and (D) the others. For instance, the taxa such as *Thaumarchaeota* and *Nitrospirae* were in the category A. The taxa such as *Alphaproteobacteria* and *Betaproteobacteria* were in the category B. The taxa such as *Chloroflexi* and *Deltaproteobacteria* were in the category into C.

To assess the differences in detection frequency of clones at finer taxonomic levels for the first- to fourth-most abundant taxa, i.e. *Thaumarchaeota* (in particular, *Nitrosopumulaceae*), *Alpha-*, *Delta-*, and *Gammaproteobacteria*, these taxa were divided into several groups based on the phylogenetic trees: a, e–z–q, and h–k–u for *Nitrosopumulaceae* of *Thaumarchaeota*; Alpha-I to -VI for *Alphaproteobacteria*; Delta-I to -VII for *Deltaproteobacteria*; and Gamma-I to -XI for *Gammaproteobacteria* (S3A–S3C and S3I Fig). The grouping for *Nitrosopumulaceae* was based on previous papers ([36] and references therein). The groupings for the proteobacterial classes are defined in the present study, and roughly (but not exactly) correspond to the phylogeny of the family or order level (S3A–S3C Fig). The differences in the presence/absence of clones in each group and each clone’s relative abundance (Fig 2B) also reflect the observed differences in the PCoA. As following the above categorization, for instance, the taxa such as a and e–z–q groups of *Nitrosopumulaceae*, Alpha-I and -V, Delta-I and -IV, and Gamma-II and -X were in the category A. In contrast, the taxa such as Alpha-VI, Delta-V, Gamma-V, and -VI were in the category B.

More than half of the crust communities (representing the libraries MnTk14, MnTk30, MnRy12, MnRy21, and MnDi18) clustered apart from the sediment communities (Fig 1). The other crust communities (representing the libraries MnTk12, MnTk22, and MnDi15) were relatively similar to the sediment communities. However, we could not clearly find a difference in the presence/absence of clones in each taxa/group and their relative abundance between the former five and the latter three crust libraries in the present study.

#### Shared members among crusts

Venn diagrams show the numbers of 16S rRNA gene OTUs shared among habitat types (i.e. crust, sediment, seawater)(S5A Fig), among regions (Takuyo-Daigo Seamount, Ryusei Seamount, Daito Ridge) for the crust members (S5B Fig), and among water depths (from 1200 m to 3000 m) for the crust members of the Takuyo-Daigo Seamount (S5C Fig). Among the habitat types (S5A Fig), only a small proportion (2.1% and 1.1%, respectively) of the total OTUs of the crust samples and those of the sediment samples were also present in the seawater samples (4.6% and 2.0% of the total OTUs of the seawater samples were shared with the OTUs detected in the crusts and with the sediments). A similar trend was observed for each depth and region (S5A Fig). These results are consistent with the difference between the seawater samples and the crust and sediment samples observed in the PCoA (Fig 1). Ten OTUs were common to the crust members of all regions, and those OTUs were affiliated with Alpha-I and -V, Gamma-II, Delta-IV, *Actinobacteria*, *Nitrospirae*, and *Nitrosopumulaceae* of *Thaumarchaeota* (a, e–z–q, and h–k–u)(S5B Fig). Five OTUs were common to the crust members for all four depths of the Takuyo-Daigo Seamount, and were affiliated with Gamma-IX, Delta-IV, and *Nitrosopumulaceae* (e–z–q and h–k–u)(S5C Fig).

**Fig 3:**
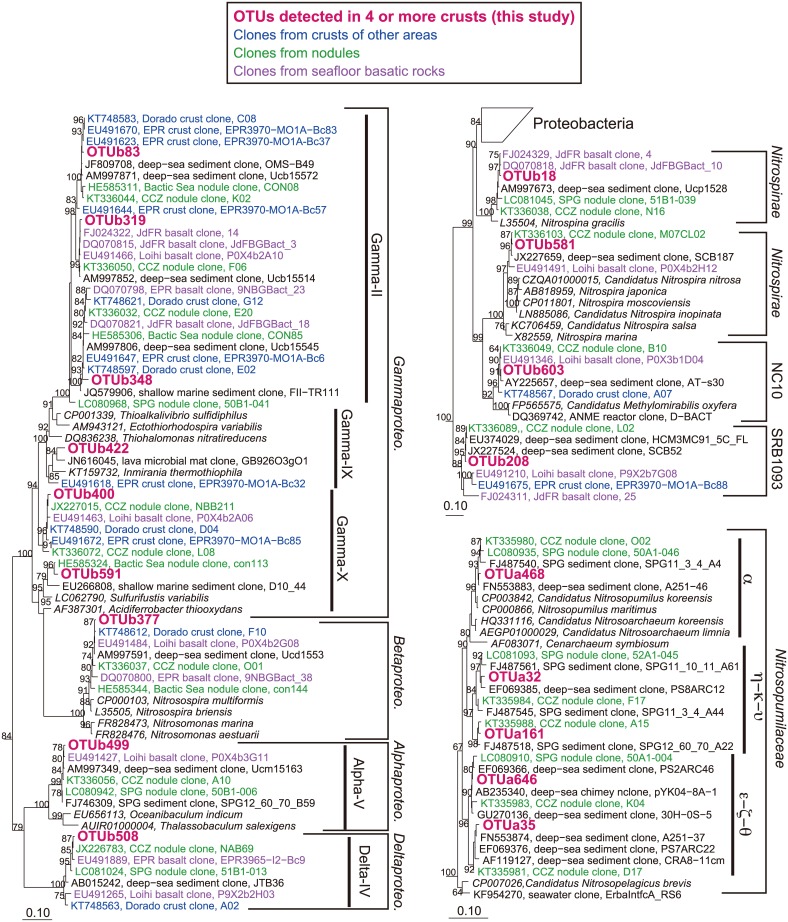
Phylogenetic trees of commonly detected OTUs in the crust samples. The 18 OTUs detected in the present study (Table 2) were in bold and red color. Clones recovered from crusts, nodules, and oceanic basalts were colored in blue, green, and purple, respectively. Bootstrap values (>50 of 100 replicates) are shown at the branch points. The scale bar represents 0.1 nucleotide substitutions per sequence position. The groupings for *Nitrosopumulaceae*, *Alpha*-, *Delta*-, and *Gammaproteobacteria* are based on the phylogenetic trees (S3A–S3C and S3I Fig).

Regardless of depth and region, 18 OTUs were common to 4 or more of all the crust samples (total = 8; Table 2). A phylogenetic tree for the 18 OTUs and closely related clones is shown in Fig 3. Out of the 18 OTUs, six OTUs were affiliated with *Gammaproteobacteria* including Gamma-II, -IX, and–X; five were affiliated with *Nitrosopumulaceae* (a, e–z–q, and h–k–u); and a single OTU was affiliated with Delta-IV of *Deltaproteobacteria*, *Nitrosomonadales* of *Betaproteobacteria*, Alpha-V of *Alphaproteobacteria*, *Nitrospirae*, *Nitrospinae*, NC10, or SBR1093. Notably, the OTUa646 in *Nitrosopumulaceae* (e–z–q) was detected in seven of eight crust samples from each depth and region (S5B and S5C Fig, Table 2) and its detection frequencies ranged from 2.4% to 12.5% of the total numbers of clones for each library (average±SD, 5.7±3.8%). It was also found in five of six sediment samples (detection frequency range: 1.1–9.3%; average±SD, 5.0±3.4%), but was not detected in the seawater samples. Three of the 18 OTUs (affiliated with Delta-IV, Gamma-IX and -X) were detected only in the crust samples.

**Table 2:** OTUs of commonly detected in the ferromanganese crusts and nodules.

OTU ID	Taxa	Number of the detected samples			Crust^a^			Nodule^a^			
		Mn crust	Sediment	Seawater	EPR	Dorado		CCZ	Baltic Sea	SPG	
		(/8 samples)	(/6 samples)	(/6 samples)	(2612 m)	(3007–3039 m)		(4107–4253 m)	(54–62 m)	(4852–5697 m)	
OTUb499	*Alphaproteobacteria*; Alpha-V	4	2	0	N.D.	N.D.	Otu000029	KT336056	N.D.	LC080942	SRX301844
OTUb377	*Betaproteobacteria*; *Nitrosomonadales*	5	3	0	N.D.	KT748612	Otu000009	KT336037	HE585344	N.D.	SRX301969
OTUb508	*Deltaproteobacteria*; Delta-IV	5	0	0	N.D.	KT748563	Otu000027	N.D.	N.D.	LC081024	SRX301897
OTUb319	*Gammaproteobacteria*; Gamma-II	6	3	0	*EU491644*	KT748583	Otu000006	KT336050	HE585311	*LC080968*	SRX301853
OTUb348	*Gammaproteobacteria*; Gamma-II	6	5	0	EU491647	KT748597	Otu000034	KT336032	*HE585306*	N.D.	SRX301844
OTUb83	*Gammaproteobacteria*; Gamma-II	5	1	0	EU491623	KT748583	Otu000005	KT336044	HE585311	N.D.	SRX301844
OTUb422	*Gammaproteobacteria*; Gamma-IX	5	0	0	*EU491618*	N.D.	Otu001338	N.D.	N.D.	N.D.	SRX301844
OTUb591	*Gammaproteobacteria*; Gamma-X	4	0	0	N.D.	N.D.	Otu000293	N.D.	HE585324	N.D.	N.D.
OTUb400	*Gammaproteobacteria*; Gamma-X	4	5	0	EU491627	KT748590	Otu000025	*KT336072*	N.D.	N.D.	SRX301844
OTUb603	NC10	4	3	0	N.D.	KT748567	Otu000103	KT336049	N.D.	N.D.	N.D.
OTUb18	*Nitrospinae*	4	3	0	N.D.	N.D.	Otu000026	KT336038	N.D.	LC081045	SRX301860
OTUb581	*Nitrospirae*	5	1	0	N.D.	N.D.	Otu000151	KT336103	N.D.	N.D.	SRX301844
OTUb208	SBR1093	4	3	0	N.D.	N.D.	Otu000110	KT336089	N.D.	N.D.	SRX301844
OTUa468	*Thaumarchaeota*; a	5	6	0	N.A.	N.D.	Otu000001	KT335980	N.A.	LC080935	SRX301983
OTUa646	*Thaumarchaeota*; ε−ζ−θ	7	5	0	N.A.	N.D.	Otu000249	KT335983	N.A.	LC080910	SRX301983
OTUa35	*Thaumarchaeota*; ε−ζ−θ	4	4	0	N.A.	N.D.	Otu000271	KT335981	N.A.	N.D.	SRX301844
OTUa161	*Thaumarchaeota*; η−κ−υ	5	3	0	N.A.	N.D.	Otu000042	KT335988	N.A.	LC081073	SRX301969
OTUa32	*Thaumarchaeota*; η−κ−υ	5	5	0	N.A.	N.D.	Otu000067	KT335984	N.A.	LC081093	SRX301844
	Reference	This study; Nitahara *et al*. (2011)			Santelli *et al*. (2008)	Lee *et al*. (2015)		Blöthe *et al*. (2015)	Yli-Hemminki *et al*. (2014)	Shiraishi *et al*. (2016)	Tully and Heidelberg (2013)

^a^ Sampling area (CCZ, Clarion and Clipperton Zone; SPG, South Pacific Gyre; EPR, East Pacific Rise), water depth, and representative environmental sequences (SRX or Otu of the head letters for experiment ID of pyrosequencing or OTU ID for Miseq tags provided by the authors, the others for accession numbers of clones of Sanger sequencing) with >97% similarity (or 95% similarity in italics) are shown. N.D., not detected. N.A., not analyzed.

### amoA gene clone libraries

To detect ammonia oxidizers and investigate their phylogenetic diversity and community structure, we performed PCR clone analyses targeting *amoA* genes encoding the alpha subunit of ammonia monooxygenase, a key enzyme in ammonia oxidation. The results are summarized in S2 Table. Lists of the bacterial and archaeal *amoA* gene clones corresponding to each OTU are shown in S5 and S6 Tables. Phylogenetic trees for all OTUs are given in S6 Fig.

**Fig 4:**
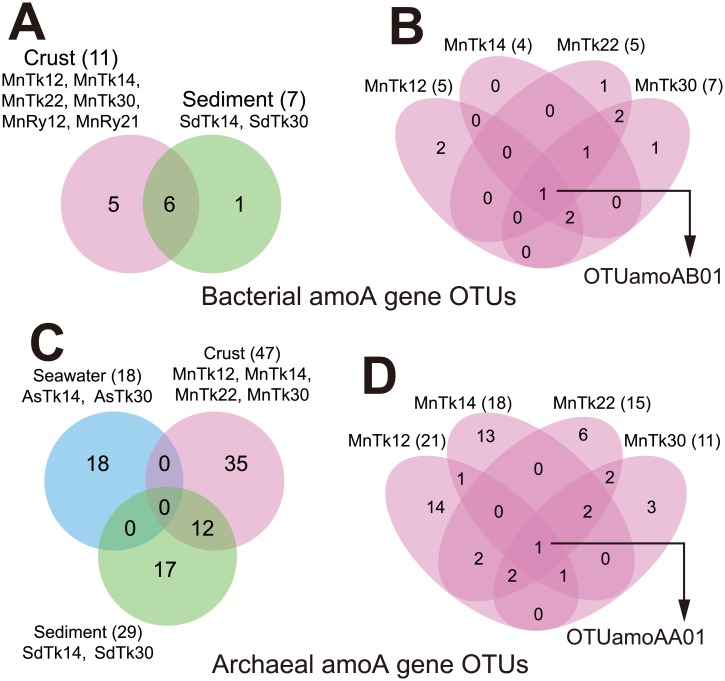
Venn diagrams showing numbers of unique and shared OTUs of *amoA* genes for the crust samples of the Takuyo-Daigo Seamount. Comparison of bacterial *amoA* OTUs (A) among habitat types and (B) among water depths, and archaeal *amoA* OTUs (C) among habitat types and (D) among water depths are shown. Numbers in circles and in parentheses indicate the numbers of OTUs. Names of the common OTUs among them are shown.

Bacterial *amoA* genes were detected in the crust and sediment samples, but not in the seawater samples. The alpha diversity of the bacterial *amoA* in the crust libraries was comparable to or higher than that in the sediment libraries (S2 Table). Coverage values were 81.8% or higher, indicating that a majority of clones in the libraries could be recovered, as supported by the nearly saturated rarefaction curves (S7 Fig). Over half of the crust OTUs and most of the sediment OTUs were in common (Fig 4A). Most of the OTUs were found in all of the crust samples of the Takuyo-Daigo Seamount despite of differences in water depth (Fig 4B). All of the bacterial *amoA* OTUs were related to those of AOB in *Nitrosomonadales*, such as *Nitrosospira* spp. and *Nitrosomonas* spp. (S6A Fig). This is consistent with the detection of the 16S rRNA genes (e.g. OTUb377) related to the AOB found in the crust and sediment samples, but not in the seawater (S3B Fig).

In contrast to bacterial *amoA*, archaeal *amoA* genes were detected in all samples from all habitat types, i.e. crust, sediment, and seawater. A Venn diagram (Fig 4C) showed that none of the OTUs in the crust and sediment samples were found in the seawater samples. In fact, phylogenetic analysis revealed that the *amoA* genes detected in the crust and sediment samples are clustered into a different group from that of the seawater samples, although all groups are classified in Thaumarchaeota AOA group (S6B Fig). The clustering of the archaeal *amoA* (LAC and HAC, for low and high ammonia concentration AOA groups, respectively) was reported in a previous study [37]. This result is clearly consistent with the archaeal 16S rRNA gene phylogeny (S3I Fig) showing the presence of the shared cluster detected both in the crust and sediment samples but not the seawater samples. Another Venn diagram (Fig 4D) shows that 40.0% or less of OTUs in the crust samples collected at deeper depths (2200 m and 3000 m) were unique for each depth, whereas 66.7% or more of OTUs in the shallower samples (1200 m and 1400 m) were unique for each depth.

## Discussion

### Limitations of the molecular analysis

We are able to address the common members in the archaeal and bacterial communities for each habitat that were apparent regardless water depth or regions. It should be noted, however, that one limitation of our PCR clone analyses is the small numbers of analyzed clones (*c*. 100 or less) for each sample for both of 16S rRNA genes and *amoA* genes, resulting in low coverage values (S2 Table) and unsaturated rarefaction curves (S4 and S7 Figs). These results suggest that we have detected only the relatively abundant members in the communities, and that the numbers of shared members shown in the present study is still underestimated. In addition, we could not draw conclusions about community differences due to water depth in a region or among regions at a particular depth. Specifically, the number of samples and clones were too low to differentiate physicochemical differences (such as differences in the dependence on DO concentration) at different water depths or to differentiate geographical differences (i.e. among the regions) in PCoA (Fig 1) and the Venn diagrams (S5B and S5C Fig for 16S rRNA genes, and Fig 4B and 4D for *amoA* genes). Nevertheless, our results provide valuable insights into microbial communities on the crusts. At the very least, community differences among the habitat types reflect the different environmental conditions associated with the habitats when six to eight samples were combined for each habitat (containing 444–668 clones).

Another limitation is that our DNA-based analyses are only able to determine the presence of the detected members, but not to assess their activity. To this end, RNA-based analyses and/or in situ measurements are needed. It is also a limitation that only testing for a particular subunit of the amoA gene does not indicate full capability of this metabolism. Furthermore, the physiology of the members detected by their 16S rRNA genes cannot be determined, but rather only inferred from their phylogeny. Remarkably, most of the OTUs in the crust and sediment samples were not grouped at the genus level (93.1% and 93.3% of total number of OTUs, respectively), in contrast to those in the seawater samples (67.6%). It is difficult to even infer their physiology. Therefore, the discussion could potentially be modified if we could cultivate the not-yet-cultivated members derived from the OTUs and elucidate their physiologies.

### Comparison of microbial communities among habitats

Our comparative analysis of the sequenced microbial communities among the habitats clearly shows that the crust communities differ from the communities of the overlying bottom seawater at every depth and every region, in terms of community membership and structure, phylogenetic diversity, and cell density, suggesting that the crust community is generally indigenous at northwestern old seamounts and cannot be attributed to the simple accumulation of precipitating seawater communities. Our results of diversity analyses are consistent with those of previous reports, which showed that the alpha diversity of exposed rocks with descriptions of Mn coatings is higher than that of the surrounding seawater [7, 8]. The characteristics of the seawater communities determined in the present study are consistent with those in deep-sea bottom seawater in other Pacific areas [38, 39]. Similarly, the sediment communities also clearly differ from the seawater communities, which is consistent with other reports of oligotrophic sediments [40, 41]. Nitahara *et al*. [6] documented this similarity between the crust and sediment communities, both of which are different from the seawater communities at 3000 m water depth on the Takuyo-Daigo Seamount. The present study extends the difference to be more general horizontally (i.e. between regions) and vertically (i.e. at different water depths) in the northwestern Pacific.

The comparison of the members in the communities between the crusts and the sediments shows that a part of members is shared (Fig 4A and 4C, S5A Fig), which is consistent with their relatively similar community structures (Fig 1). Based on our strict ROV and sample-treatment protocols, we are confident that most of the shared members are indigenous for each habitat, and not a result of cross contamination. Given the similar environmental conditions at the same depths, in terms of temperature, pressure, salinity, and oxygen concentration, it is not surprising that members in communities partially overlap.

### Potential for ammonia oxidation on crusts

The detection of *amoA* genes suggests that ammonia oxidizers are commonly present in the crusts, as well as the sediments and seawater, as supported by the detection of the 16S rRNA genes related to ammonia oxidizers. Nitahara *et al*. [6] have hypothesized that ammonia is the main energy source sustaining the crust microbial ecosystem, based on the detection of only 16S rRNA genes related to ammonia oxidizers. The present study supports this hypothesis with the detection of *amoA* in the crust samples, and suggests the wide distribution of the AOA and AOB horizontally and vertically in the northwestern Pacific Ocean bottom. Nitrite produced by ammonia oxidizers can be sequentially oxidized to nitrate by nitrite oxidizers of the taxa *Nitrospirae* and *Nitrospinae* detected in the crusts and sediments. Thus, in addition to organic carbon precipitated from the overlying seawater, ammonia ubiquitous in seawater is likely to be one of the energy sources for sustaining the crust ecosystem in the northwestern Pacific. Furthermore, autotrophic (or mixotrophic) AOA and AOB can fix inorganic carbon into organic carbon that then serves as carbon and energy sources for organoheterotrophs in the ecosystem. Further analyses, such as metatranscriptomics and in situ measurement of ammonia oxidation and carbon fixation, are needed to show how AOA and AOB are functioning.

Although bacterial *amoA* genes and 16S rRNA genes related to AOB were detected in the crusts and sediments, they were not detected in the seawater in our analysis. On the other hand, AOA were detected in all of these habitats. This difference in distribution of AOB and AOA potentially reflects the difference in ammonia concentrations between the seawater and the crusts and sediments. Generally, ammonia concentrations of seawater are <5 μM, and those of crusts and sediments are likely higher due to degradation of organic compounds accumulated on the crusts and in the sediments. Cultivated AOB require ammonia concentrations of >1 μM for growth [42], but a cultivated AOA, *Nitrosopumilus maritimus*, can grow at a low ammonia concentration (<10 nM) [43]. In fact, AOB often outcompete AOA in marine sediments with higher ammonia concentrations [44]. Based on the phylogenies of the 16S rRNA and *amoA* genes, some of the detected bacterial *amoA* genes are likely to be derived from AOBs possessing the 16S rRNA genes of the previously defined *Nitrosospira* cluster I [45] and detected in oligotrophic sediments [44]. In addition to ammonia concentration, other factors, such as salinity, pH, and oxygen concentration, may also influence niche differentiation of AOA/AOB (e.g. [46–48], which might partially explain the detection of AOB only in the crust and sediment samples.

The phylogenetic difference in the AOA represented in 16S rRNA and *amoA* genes between the seawater samples and the crust and sediment samples may reflect physiological differences. Such a phylogenetic difference in AOA has been reported for marine sediments with overlying seawater, based on 16S rRNA gene and/or *amoA* gene analysis [18, 36]. Marine AOA in *Nitrosopumulaceae* have been found to vary in their pH growth range and optimum, and their utilization of organic acids [49]. Similarly, a phylogenetic difference in soil AOA has been associated with environmental conditions ([50] and references therein). In addition, the ‘scaffolding’ of the substrate available to the microbes clearly differs between the crusts and sediments (i.e. a solid surface) and the seawater (liquid). Thus, differences in life styles, i.e. surface-attached or free-living, which is generally observed in prokaryotes, and also reported in *Nitrosopumulaceae* recently [51], might also correspond to a phylogenetic difference.

Remarkably, there is a cosmopolitan AOA represented as OTUamoAA01 (detected in all six analyzed samples of the crust and sediments; Fig 4 and S6 Table), which might correspond to the archaea shown to possess the 16S rRNA gene of OTUa646, i.e. the cosmopolitan archaea of *Nitrosopumulaceae* (detected in seven of eight crust samples, and in five of six sediment samples; S5 Fig and Table 2), regardless of differences in water depth, region, and habitat type. This result suggests that the cosmopolitan AOA is probably adapted to the wide range of the environmental conditions found at different depths and in different regions, such as DO concentrations (as shown in the CTD profile, S2 Fig), and nutrients [52]. Furthermore, environmental clones closely related (>97% similarity) to OTUamoAA01 and OTUa646 have been detected in various locations including the Pacific, Atlantic, and Arctic oceans, as revealed by a survey of public databases using BLAST searches (S7 Table), suggesting that the cosmopolitan AOA are widely distributed not only in the crusts and sediments in the north-western Pacific but also throughout the world’s oceans.

### Common members in crusts and nodules

The bacteria derived from the OTUs in Gamma-IX and -X (including *Thioalkalispiraceae* with S-oxidizing bacteria (SOB) *Thioalkalispira* spp. and *Acidiferrobacteraceae* with S- and Fe-oxidizing bacteria (FeOB) *Acidiferrobacter* spp.) were detected in half or more of the crust samples. Although these OTUs show low similarity (<95%) to cultivated species, they may contribute to Fe and S cycling. The bacteria derived from the OTUs in Gamma-II, Delta-IV, Alpha-V, NC10, and SBR1093 (Table 2) were also frequently detected in the crust samples. However, because these OTUs are not closely related to any cultivated species (similarity <90%), it is difficult to even infer their metabolic function. Further investigations, such as cultivation and single-cell genomics, will provide clues to their functional roles in microbial ecosystems.

Notably, despite other researchers having employed different methodological procedures, environmental 16S rRNA gene sequences related to the commonly detected OTUs in our crusts have also been frequently detected in crusts and nodules collected in a variety of areas [7, 8, 10–13](Fig 3, Table 2). In the previous PCR clone analyses, these sequences related to the common OTUs were not detected in reference seawater samples if available in the reports [7, 8], as well as in our results. In a different study, which used next-generation sequencing analysis [7], these sequences related to the common OTUs were relatively abundant in the libraries from the crust samples but rarely or not detected in those from the seawater samples. This leads us to hypothesize that the bacteria derived from the OTUs are commonly present and relatively abundant on the surface of the Mn oxide coatings on the seafloor. Further sampling of crusts and nodules from various areas, more sequencing efforts using next generation techniques, and quantitative analyses targeting the common members will be needed to assess the hypothesis.

### Microbial contribution to the formation of crusts

Microbes are thought to contribute to the formation of crusts; however, robust evidence for this is lacking, as is an explanation of how microbes might contribute to these crusts. To date, phylogenetically diverse Mn-oxidizing bacteria (MnOB) have been reported, including members of *Bacillus*, *Pseudomonas*, *Leptothrix*, *Erythrobacter*, *Pedomicrobium*, and *Roseobacter*, with enzymatic or superoxide-mediated reactions [53, 54]. Several MnOB of *Marinobacter*, *Halomonas*, *Alteromonas*, *Pseudoalteromonas*, and *Acinetobacter* have been isolated from the Loihi Seamount, Hawaii, although it is unknown how they oxidize Mn. In the present study, the 16S rRNA genes related to MnOB of *Marinobacter* and *Pseudomonas* (Gamma-IV), *Alteromonas* and *Pseudoalteromonas* (Gamma-VI), *Acinetobacter* (Gamma-V), *Erythrobacter* (Alpha-III), and *Leptothrix* (*Betaproteobacteria*) were detected only in the seawater samples (S3C Fig). Potential MnOB related to *Pedomicrobium* (Alpha-I) was detected in the crust samples but not in the seawater samples. These putative MnOB could contribute to manganese oxidation in the crust formation. The reduction of Mn in nodules by Mn reducers related to *Shewanella* spp. has been suggested [11]. However, no 16S rRNA gene sequences closely related to Mn reducers including *Shewanella* were detected in the present study.

### Conclusion

In the present study, we collected the crusts at different water depths (1200 to 3000 m) and from different tectonic settings, in addition to samples of the surrounding oligotrophic sediments and bottom seawater for reference, and we analyzed the microbial communities using the same procedures, and then compared them. Even though the analyses had certain limitations, our results highlight the differences and commonalities in the microbial communities on the crusts, and in sediments and seawater. The comparative analysis indicated the presence of common members within the crusts, which included AOA and AOB as supported by 16S rRNA and *amoA* gene data, and potentially NOB, FeOB, and SOB as inferred only from 16S rRNA gene data, all of which may play a significant role in ecosystem functioning and geochemical cycling there. Furthermore, the combination of previous reports and our present results suggest that a subset of the commonly detected community members is widely distributed in crusts and nodules on the vast seafloor.

## Supporting information

S1 Fig Photos of the crust samples collected from the Takuyo-Daigo Seamount, Ryusei Seamount, and Daito Ridge. Surface and section surface of each sample, left and right in each photo. Some samples had bumpy surfaces. The basement rocks were basalts or limestones.
(PDF)

S2 Fig Water depth dependence on temperature (red), salinity (blue), and dissolved oxygen concentration (green) in the sampling regions, the Takuyo-Daigo Seamount, Ryusei Seamount, and Daito Ridge. The depths for the sample collections are indicated with lines and the sample IDs.
(PDF)

S3 Fig Phylogenetic trees of 16S rRNA gene OTUs. Trees for (A) *Alphaproteobacteria*; (B) *Betaproteobacteria* and *Deltaproteobacteria*; (C) *Gammaproteobacteria*; (D) *Acidobacteria*, *Gemmatimonadetes*, and *Cyanobacteria*; (E) *Actinobacteria*, *Bacteroidetes*, and *Chlorobi*; (F) *Caldithrix*, *Chlamydiae*, *Firmicutes*, *Verrucomicrobia*, *Nitrospinae*, and *Nitrospirae*; (G) *Chloroflexi*, *Planctomycetes*, and *Omnitrophica* (formally candidate division OP3); (H) other bacterial phyla and uncultured clone groups; and (I) *Archaea*, are shown. Numbers in parentheses following the OTU name indicate the numbers of clones from the crust libraries (red), the sediment libraries (green), and the seawater libraries (blue). Cultured species with a black star indicate Mn-oxidizing bacteria in (A) and (B). OTUs with a filled black circle indicate the common members among the crusts (see Table 2). Environmental clones recovered from crusts and oceanic basalts are colored brown.
(PDF)

S4 Fig Comparison of Chao1 species richness estimates, Shannon diversity index, and rarefaction curves among the sample types. Data for the crusts (red), sediments (green), and seawater samples (blue) are shown. Box plots are used for the Chao1 species richness estimates and Shannon diversity index. *, p <0.001; **, p <0.01.
(PDF)

S5 Fig Venn diagrams showing numbers of unique and shared OTUs of 16S rRNA genes. (A) Among habitat types, (B) among regions for the crust samples, and (C) among water depths for the crust samples of the Takuyo-Daigo Seamount. Numbers in circles and in parentheses are the numbers of OTUs. The names and taxonomic affiliations of the common OTUs among them are indicated. The last two letters of the sample name indicate the approximate sampling depth in hundred meters. Numbers in circles and in parentheses are the numbers of OTUs. The last two letters of the sample name indicate the approximate sampling depth in hundred meters.
(PDF)

S6 Fig Phylogenetic trees of *amoA* gene OTUs. Trees for (A) bacterial and (B) archaeal *amoA* genes are shown. OTUs in brown and purple indicate those from oceanic basalts from a previous study and from crusts in the present study, respectively. Numbers in parentheses following the OTU name indicate the numbers of clones from the crust libraries (red), the sediment libraries (green), and the seawater libraries (blue).
(PDF)

S7 Fig Rarefaction curves for *amoA* gene clone libraries. Red, green, and blue lines are for the crusts, sediments, and seawater, respectively.
(PDF)

S1 Table Results of qPCR. (XLSX)

S2 Table Summary of PCR clone libraries. (XLSX)

S3 Table List of 16S rRNA gene clones for each OTU. (XLSX)

S4 Table p values by p-test (lower) and UniFrac significance test (upper) between two samples. (XLSX)

S5 Table List of bacterial amoA clones for each OTU. (XLSX)

S6 Table List of archaeal amoA clones for each OTU. (XLSX)

S7 Table Environmental clones with 97% or higher similarity with OTUa646 or OTUamoAA01. (XLSX)
