# Supplementary figures and images for: Archaeal and bacterial communities in deep-sea hydrogenetic ferromanganese crusts on old seamounts of the northwestern Pacific
Source: PLoS One. 2017 Feb 24;12(2):e0173071. doi: 10.1371/journal.pone.0173071 (PMC5325594; doi:10.1371/journal.pone.0173071)

## Takuyo-Daigo Seamount

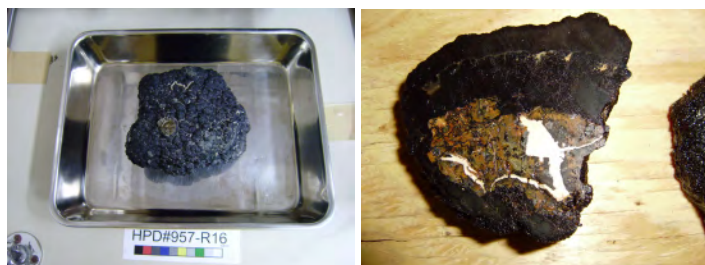

MnTk12

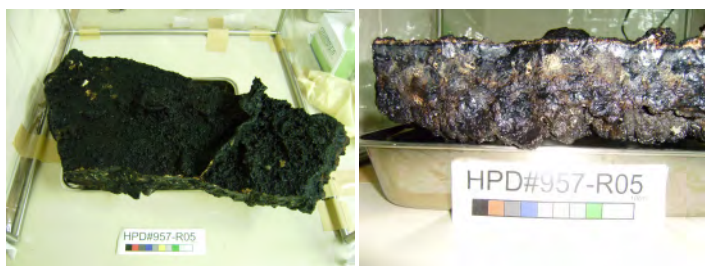

MnTk14

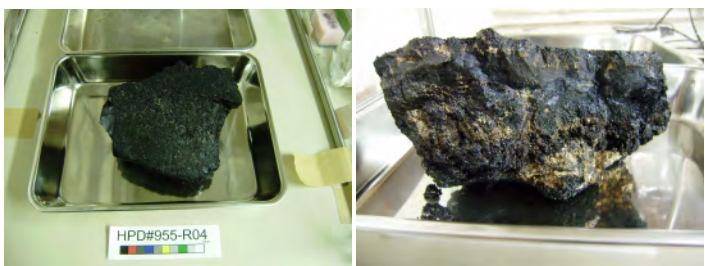

MnTk22

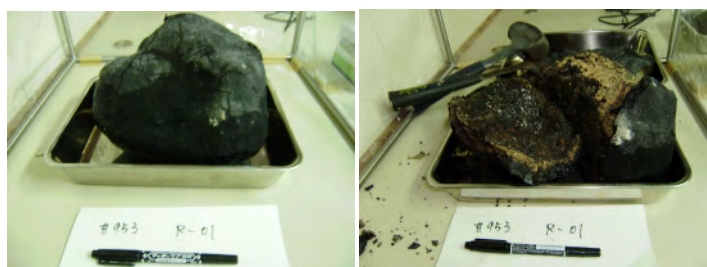

MnTk30

## Ryusei Seamount

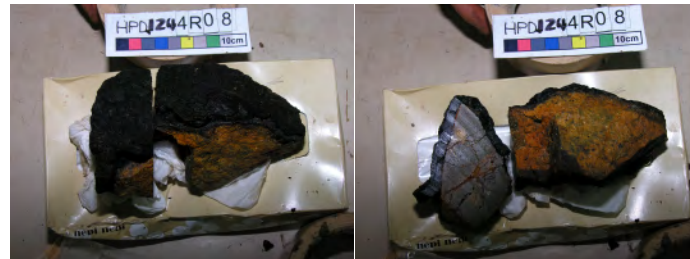

MnRy12

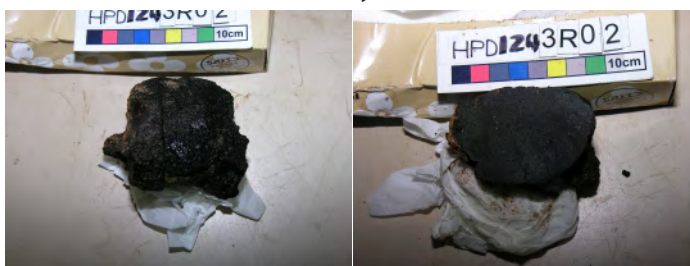

MnRy21

## Daito Ridge

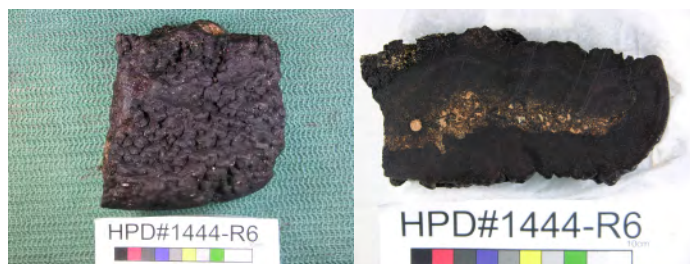

MnDi15

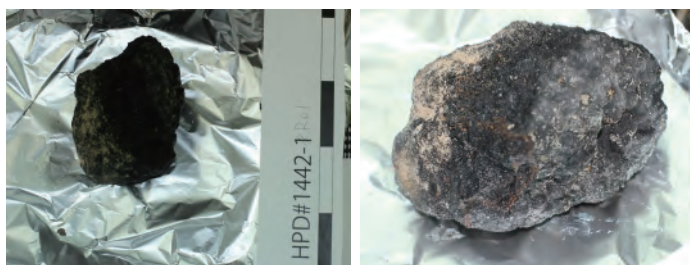

MnDi18

Fig. S1

Supplement: S1 Fig — Surface and section surface of each sample, left and right in each photo. Some samples had bumpy surfaces. The basement rocks were basalts or limestones. (PDF) [file pone.0173071.s001.pdf]

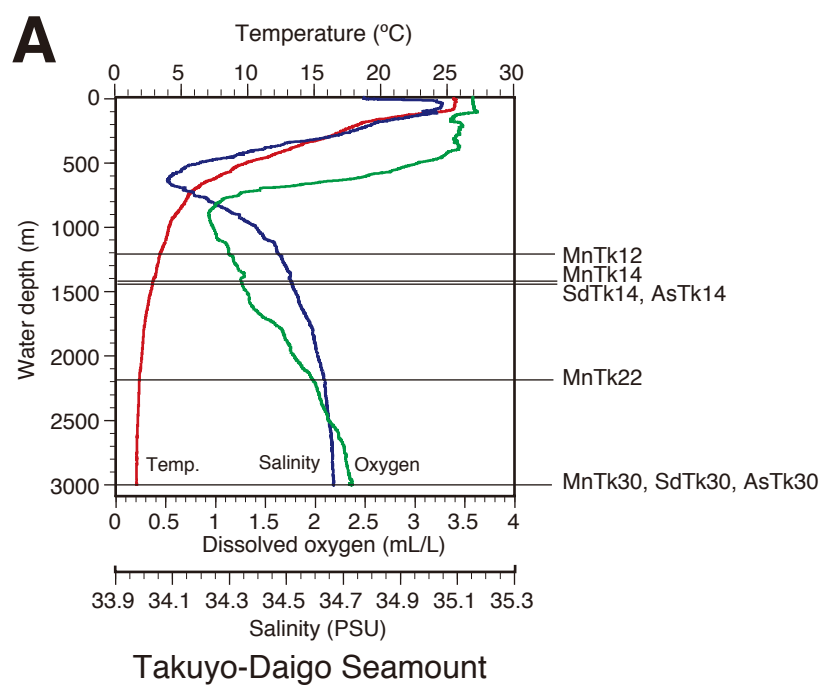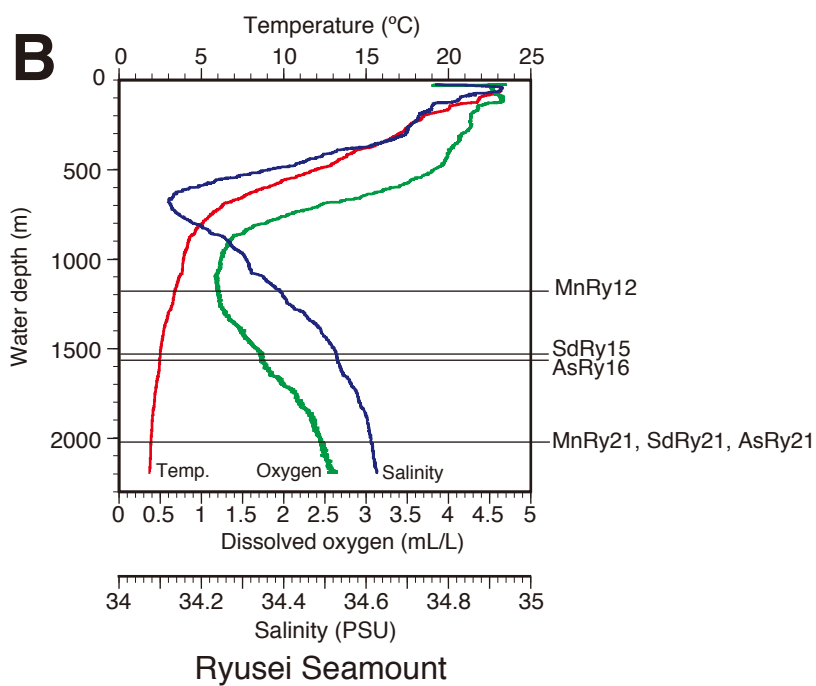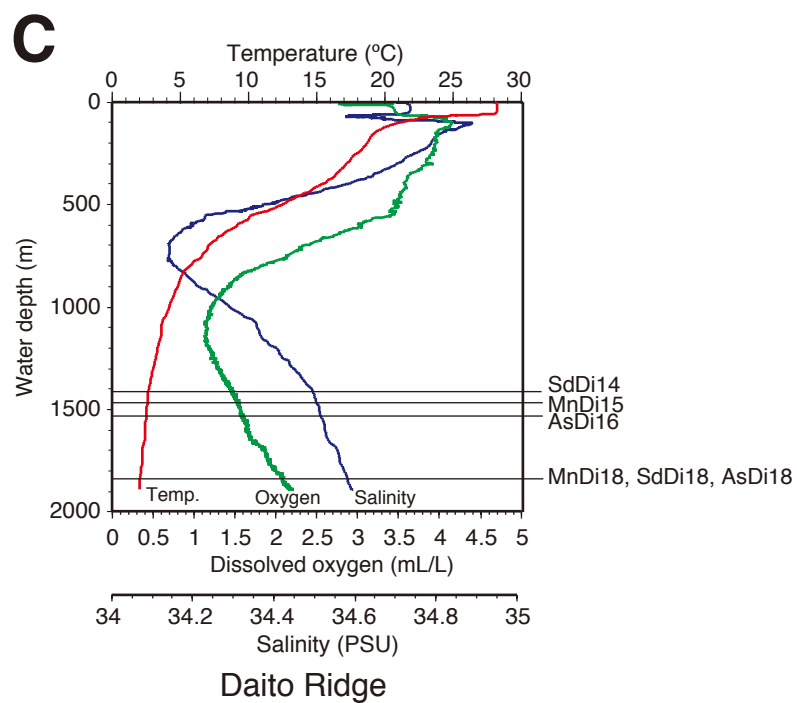

Fig. S2

Supplement: S2 Fig — The depths for the sample collections are indicated with lines and the sample IDs. (PDF) [file pone.0173071.s002.pdf]

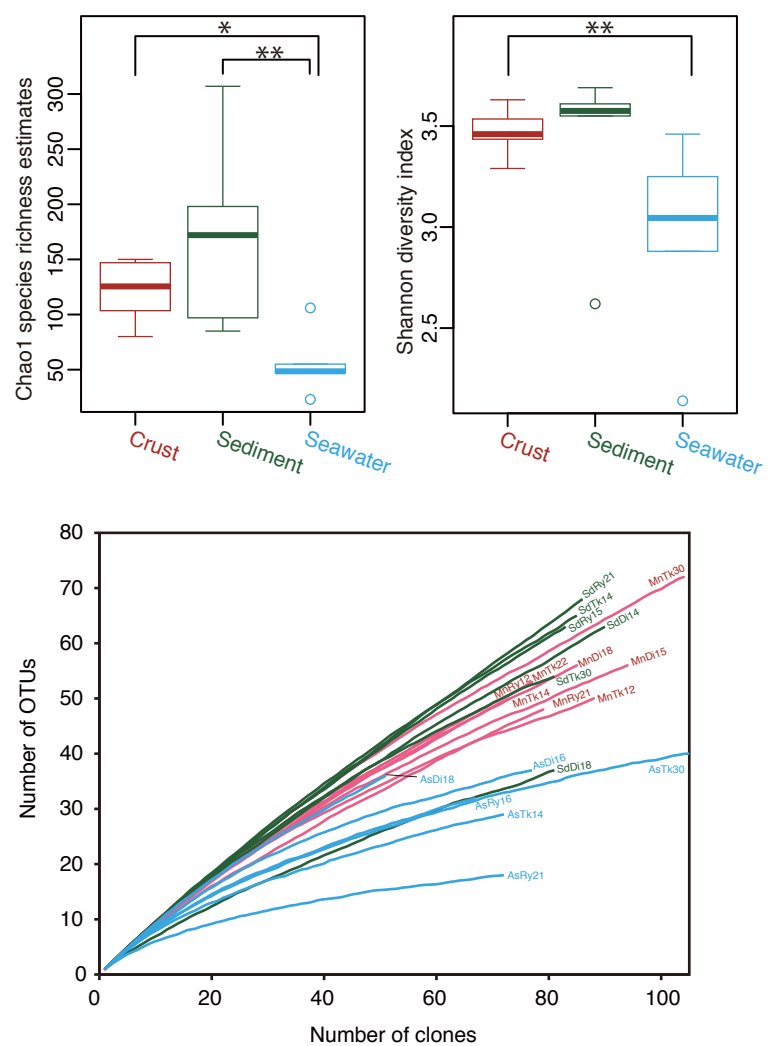

Fig. S4

Supplement: S4 Fig — Data for the crusts (red), sediments (green), and seawater samples (blue) are shown. Box plots are used for the Chao1 species richness estimates and Shannon diversity index. *, p <0.001; **, p <0.01. (PDF) [file pone.0173071.s004.pdf]

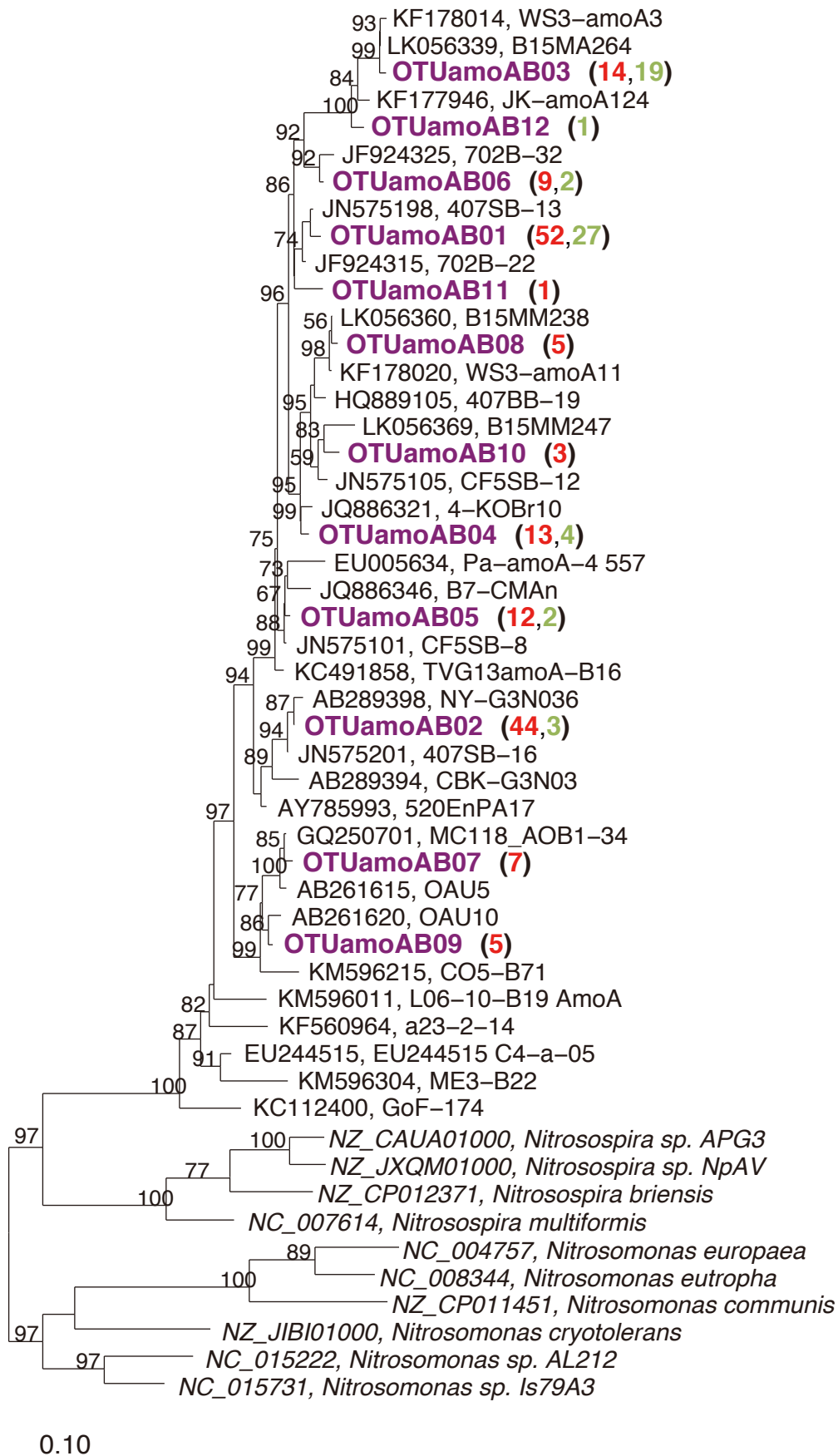

Fig. S6A

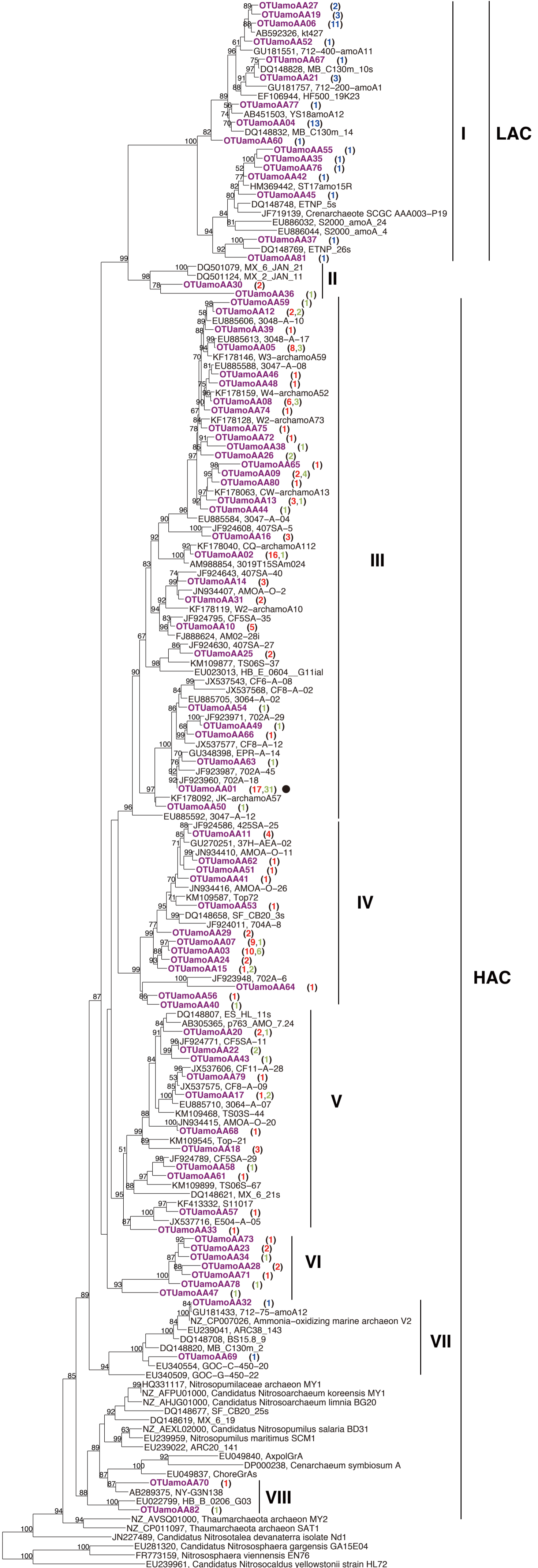

Fig. S6B

Supplement: S6 Fig — Trees for (A) bacterial and (B) archaeal amoA genes are shown. OTUs in brown and purple indicate those from oceanic basalts from a previous study and from crusts in the present study, respectively. Numbers in parentheses following the OTU name indicate the numbers of clones from the crust libraries (red), the sediment libraries (green), and the seawater libraries (blue). (PDF) [file pone.0173071.s006.pdf]

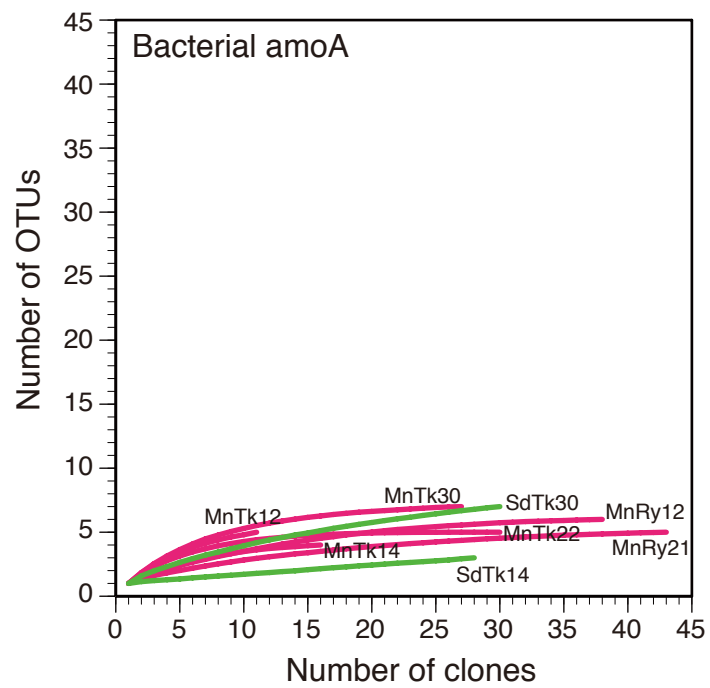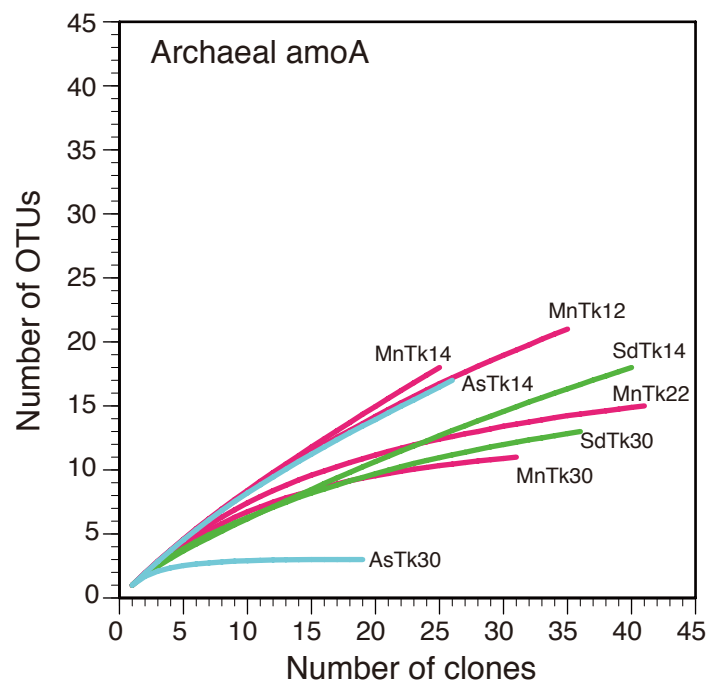

Fig. S7

Supplement: S7 Fig — Red, green, and blue lines are for the crusts, sediments, and seawater, respectively. (PDF) [file pone.0173071.s007.pdf]
