# Supplementary material for: Archaeal and bacterial communities in deep-sea hydrogenetic ferromanganese crusts on old seamounts of the northwestern Pacific
Source: PLoS One. 2017 Feb 24;12(2):e0173071. doi: 10.1371/journal.pone.0173071 (PMC5325594; doi:10.1371/journal.pone.0173071)
Supplement: S3 Fig — Trees for (A) Alphaproteobacteria; (B) Betaproteobacteria and Deltaproteobacteria; (C) Gammaproteobacteria; (D) Acidobacteria, Gemmatimonadetes, and Cyanobacteria; (E) Actinobacteria, Bacteroidetes, and Chlorobi; (F) Caldithrix, Chlamydiae, Firmicutes, Verrucomicrobia, Nitrospinae, and Nitrospirae; (G) Chloroflexi, Planctomycetes, and Omnitrophica (formally candidate division OP3); (H) other bacterial phyla and uncultured clone groups; and (I) Archaea, are shown. Numbers in parentheses following the OTU name indicate the numbers of clones from the crust libraries (red), the sediment libraries (green), and the seawater libraries (blue). Cultured species with a black star indicate Mn-oxidizing bacteria in (A) and (B). OTUs with a filled black circle indicate the common members among the crusts (see Table 2). Environmental clones recovered from crusts and oceanic basalts are colored brown. (PDF) [file pone.0173071.s003.pdf]

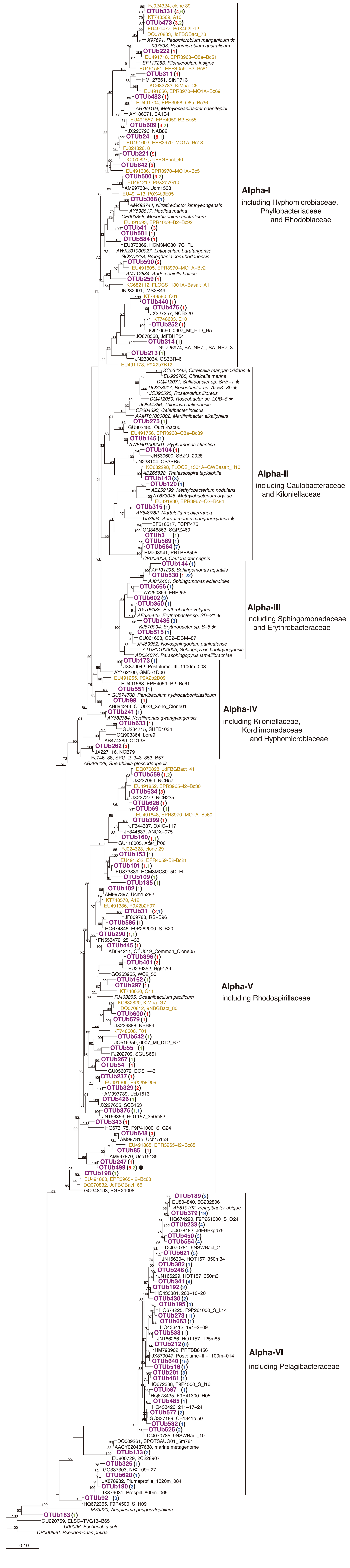
Alpha-I
including Hyphomicrobiaceae, Phyllobacteriaceae and Rhodobiaceae
Alpha-II
including Caulobacteraceae and Kiloniellaceae
Alpha-III
including Sphingomonadaceae and Erythrobacteraceae
Alpha-IV
including Kiloniellaceae, Kordiimonadaceae and Hyphomicrobiaceae
Alpha-V
including Rhodospirillaceae
Alpha-VI
including Pelagibacteraceae
0.10

Fig. S3A

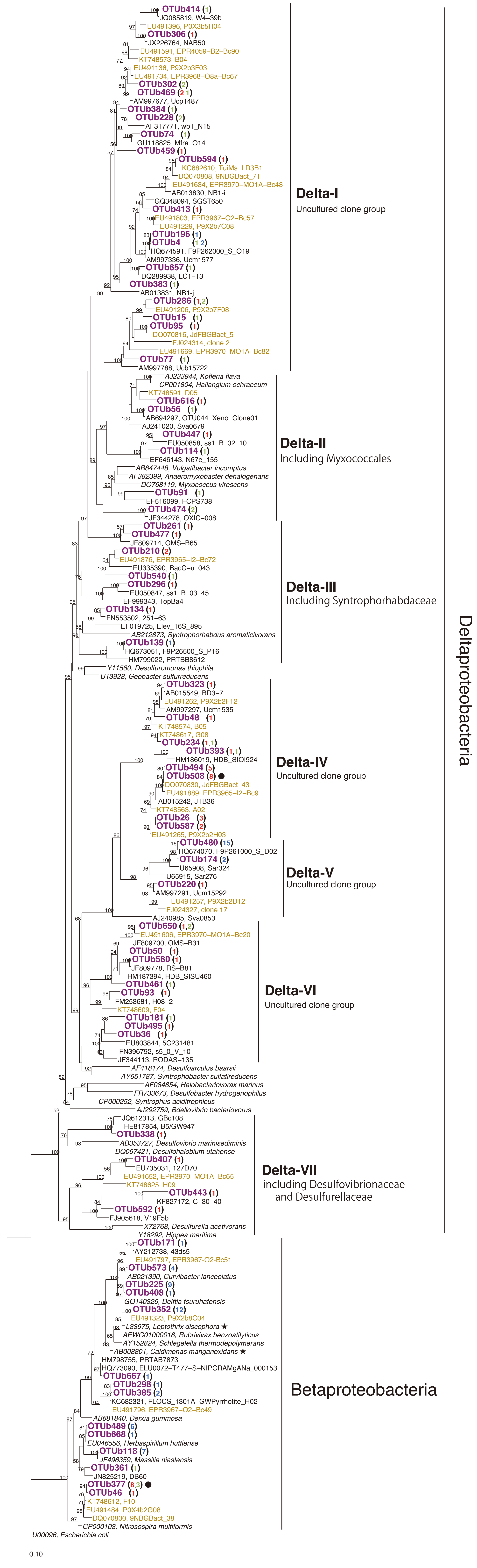
OTUb414 (1)
JQ085819, W4–39b
EU491396, P0X3b5H04
OTUb306 (1)
JX226764, NAB50
EU491591, EPR4059–B2–Bc90
KT748573, B04
EU491136, P9X2b3F03
EU491734, EPR3968–O8a–Bc67
OTUb302 (2)
OTUb469 (2,1)
AM997677, Ucp1487
OTUb384 (1)
OTUb228 (2)
AF317771, wb1_N15
OTUb74 (1)
GU118825, Mfra_O14
OTUb459 (1)
OTUb594 (1)
KC682610, TuiMs_LR3B1
DQ070808, 9NBGBact_71
EU491634, EPR3970–MO1A–Bc48
AB013830, NB1-i
GQ348094, SGST650
OTUb413 (1)
EU491803, EPR3967–O2–Bc57
EU491229, P9X2b7C08
OTUb196 (1)
OTUb4 (1,2)
HQ674591, F9P262000_S_O19
AM997336, Ucm1577
OTUb657 (1)
DQ289938, LC1–13
OTUb383 (1)
AB013831, NB1-j
OTUb286 (1,2)
EU491206, P9X2b7F08
OTUb15 (1)
OTUb95 (1)
DQ070816, JdFBGBact_5
FJ024314, clone 2
EU491669, EPR3970–MO1A–Bc82
OTUb77 (1)
AM997788, Ucb15722
Delta-I
Uncultured clone group
AJ233944, Kofleria flava
CP001804, Haliangium ochraceum
KT748591, D05
OTUb616 (1)
OTUb56 (1)
AB694297, OTU044_Xeno_Clone01
AJ241020, Sva0679
OTUb447 (1)
EU050858, ss1_B_02_10
OTUb114 (1)
EF646143, N67e_155
AB847448, Vulgatibacter incomptus
AF382399, Anaeromyxobacter dehalogenans
DQ768119, Myxococcus virescens
OTUb91 (1)
EF516099, FCPS738
OTUb474 (2)
JF344278, OXIC–008
Delta-II
Including Myxococcales
OTUb261 (1)
OTUb477 (1)
JF809714, OMS–B65
OTUb210 (2)
EU491876, EPR3965–I2–Bc72
EU335390, BacC–u_043
OTUb540 (1)
OTUb296 (1)
EU050847, ss1_B_03_45
EF999343, TopBa4
OTUb134 (1)
FN553502, 251–63
EF019725, Elev_16S_895
AB212873, Syntrophorhabdus aromaticivorans
OTUb139 (1)
HQ673051, F9P26500_S_P16
HM799022, PRTBB8612
Delta-III
Including Syntrophorhabdaceae
Y11560, Desulfuromonas thiophila
U13928, Geobacter sulfurreducens
OTUb323 (1)
AB015549, BD3–7
EU491262, P9X2b2F12
AM997297, Ucm1535
OTUb48 (1)
KT748574, B05
KT748617, G08
OTUb234 (1,1)
OTUb393 (1,1)
HM186019, HDB_SIOI924
OTUb494 (5)
OTUb508 (8)
DQ070830, JdFBGBact_43
EU491889, EPR3965–I2–Bc9
AB015242, JTB36
KT748563, A02
OTUb26 (3)
OTUb587 (2)
EU491265, P9X2b2H03
Delta-IV
Uncultured clone group
OTUb480 (15)
HQ674070, F9P261000_S_D02
OTUb174 (2)
U65908, Sar324
U65915, Sar276
OTUb220 (1)
AM997291, Ucm15292
EU491257, P9X2b2D12
FJ024327, clone 17
Delta-V
Uncultured clone group
AJ240985, Sva0853
OTUb650 (1,2)
EU491606, EPR3970–MO1A–Bc20
JF809700, OMS–B31
OTUb50 (1)
OTUb580 (1)
JF809778, RS–B81
HM187394, HDB_SISU460
OTUb461 (1)
OTUb93 (1)
FM253681, H08–2
KT748609, F04
OTUb181 (1)
OTUb495 (1)
OTUb36 (1)
EU803844, 5C231481
FN396792, s5_0_V_10
JF344113, RODAS–135
Delta-VI
Uncultured clone group
AF418174, Desulfoarculus baarsii
AY651787, Syntrophobacter sulfatireducens
AF084854, Halobacteriovorax marinus
FR733673, Desulfobacter hydrogenophilus
CP000252, Syntrophus aciditrophicus
AJ292759, Bdellovibrio bacteriovorus
JQ612313, GBc108
HE817854, B5/GW947
OTUb338 (1)
AB353727, Desulfovibrio marinisediminis
DQ067421, Desulfohalobium utahense
OTUb407 (1)
EU735031, 127D70
EU491652, EPR3970–MO1A–Bc65
KT748625, H09
OTUb443 (1)
KF827172, C–30–40
OTUb592 (1)
FJ905618, V19F5b
X72768, Desulfurella acetivorans
Y18292, Hippea maritima
Delta-VII
including Desulfovibrionaceae
and Desulfurellaceae
Deltaproteobacteria
OTUb171 (1)
AY212738, 43ds5
EU491797, EPR3967-O2-Bc51
OTUb573 (4)
AB021390, Curvibacter lanceolatus
OTUb225 (9)
OTUb408 (1)
GQ140326, Delftia tsuruhatensis
OTUb352 (12)
EU491323, P9X2b8C04
L33975, Leptothrix discophora ★
AEWG01000018, Rubrivivax benzoatilyticus
AY152824, Schlegelella thermodepolymerans
AB008801, Caldimonas manganoxidans ★
HM798755, PRTAB7873
HQ773090, ELU0072–T477–S–NIPCRAMgANa_000153
OTUb667 (1)
OTUb298 (1)
OTUb385 (2)
KC682321, FLOCS_1301A–GWPyrrhotite_H02
EU491796, EPR3967–O2–Bc49
AB681840, Derxia gummosa
OTUb489 (6)
OTUb668 (1)
EU046556, Herbaspirillum huttiense
OTUb118 (7)
JF496359, Massilia niastensis
OTUb361 (1)
JN825219, DB60
OTUb377 (8,3)
OTUb46 (1)
KT748612, F10
EU491484, P0X4b2G08
DQ070800, 9NBGBact_38
CP000103, Nitrosospira multiformis
Betaproteobacteria
U00096, Escherichia coli
0.10

Fig. S3B

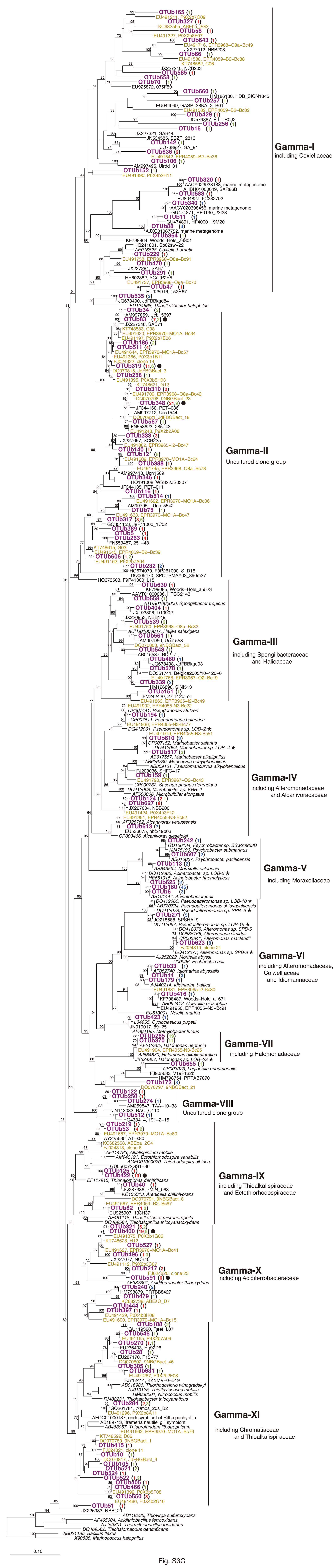
Gamma-I
including Coxiellaceae
Gamma-II
Uncultured clone group
Gamma-III
including Spongiibacteraceae and Halieaceae
Gamma-IV
including Alteromonadaceae and Alcanivoracaceae
Gamma-V
including Moraxellaceae
Gamma-VI
including Alteromonadaceae, Colwelliaceae and Idiomarinaceae
Gamma-VII
including Halomonadaceae
Gamma-VIII
Uncultured clone group
Gamma-IX
including Thioalkalispiraceae and Ectothiorhodospiraceae
Gamma-X
including Acidiferrobacteraceae
Gamma-XI
including Chromatiaceae and Thioalkalispiraceae
0.10

Fig. S3C

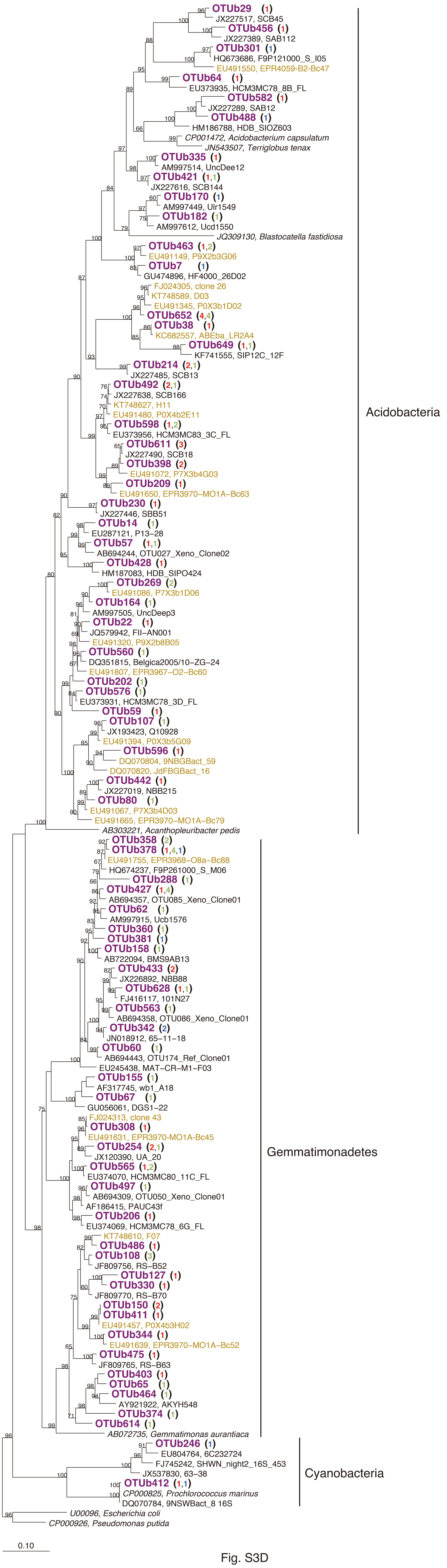

OTUb29 (1)
JX227517, SCB45
OTUb456 (1)
JX227389, SAB112
OTUb301 (1)
HQ673686, F9P121000_S_I05
EU491550, EPR4059-B2-Bc47
OTUb64 (1)
EU373935, HCM3MC78_8B_FL
OTUb582 (1)
JX227289, SAB12
OTUb488 (1)
HM186788, HDB_SIOZ603
CP001472, Acidobacterium capsulatum
JN543507, Terriglobus tenax
OTUb335 (1)
AM997514, UncDee12
OTUb421 (1,1)
JX227616, SCB144
OTUb170 (1)
AM997449, Ulr1549
OTUb182 (1)
AM997612, Ucd1550
JQ309130, Blastocatella fastidiosa
OTUb463 (1,2)
EU491149, P9X2b3G06
OTUb7 (1)
GU474896, HF4000_26D02
FJ024305, clone 26
KT748589, D03
EU491345, P0X3b1D02
OTUb652 (4,4)
OTUb38 (1)
KC682557, ABEba_LR2A4
OTUb649 (1,1)
KF741555, SIP12C_12F
OTUb214 (2,1)
JX227485, SCB13
OTUb492 (2,1)
JX227638, SCB166
KT748627, H11
EU491480, P0X4b2E11
OTUb598 (1,2)
EU373956, HCM3MC83_3C_FL
OTUb611 (3)
JX227490, SCB18
OTUb398 (2)
EU491072, P7X3b4G03
OTUb209 (1)
EU491650, EPR3970-MO1A-Bc63
OTUb230 (1)
JX227446, SBB51
OTUb14 (1)
EU287121, P13-28
OTUb57 (1,1)
AB694244, OTU027_Xeno_Clone02
OTUb428 (1)
HM187083, HDB_SIPO424
OTUb269 (2)
EU491086, P7X3b1D06
OTUb164 (1)
AM997505, UncDeep3
OTUb22 (1)
JQ579942, FII-AN001
EU491320, P9X2b8B05
OTUb560 (1)
DQ351815, Belgica2005/10-ZG-24
EU491807, EPR3967-O2-Bc60
OTUb202 (1)
OTUb576 (1)
EU373931, HCM3MC78_3D_FL
OTUb59 (1)
OTUb107 (1)
JX193423, Q10928
EU491394, P0X3b5G09
OTUb596 (1)
DQ070804, 9NBGBact_59
DQ070820, JdFBGBact_16
OTUb442 (1)
JX227019, NBB215
OTUb80 (1)
EU491067, P7X3b4D03
EU491665, EPR3970-MO1A-Bc79
AB303221, Acanthopleuribacter pedis
Acidobacteria
OTUb358 (2)
OTUb378 (1,4,1)
EU491755, EPR3968-O8a-Bc88
HQ674237, F9P261000_S_M06
OTUb288 (1)
OTUb427 (1,4)
AB694357, OTU085_Xeno_Clone01
OTUb62 (1)
AM997915, Ucb1576
OTUb360 (1)
OTUb381 (1)
OTUb158 (1)
AB722094, BMS9AB13
OTUb433 (2)
JX226892, NBB88
OTUb628 (1,1)
FJ416117, 101N27
OTUb563 (1)
AB694358, OTU086_Xeno_Clone01
OTUb342 (2)
JN018912, 65-11-18
OTUb60 (1)
AB694443, OTU174_Ref_Clone01
EU245438, MAT-CR-M1-F03
OTUb155 (1)
AF317745, wb1_A18
OTUb67 (1)
GU056061, DGS1-22
FJ024313, clone 43
OTUb308 (1)
EU491631, EPR3970-MO1A-Bc45
OTUb254 (2,1)
JX120390, UA_20
OTUb565 (1,2)
EU374070, HCM3MC80_11C_FL
OTUb497 (1)
AB694309, OTU050_Xeno_Clone01
AF186415, PAUC43f
OTUb206 (1)
EU374069, HCM3MC78_6G_FL
KT748610, F07
OTUb486 (1)
OTUb108 (3)
JF809756, RS-B52
OTUb127 (1)
OTUb330 (1)
JF809770, RS-B70
OTUb150 (2)
OTUb411 (1)
EU491457, P0X4b3H02
OTUb344 (1)
EU491639, EPR3970-MO1A-Bc52
OTUb475 (1)
JF809765, RS-B63
OTUb403 (1)
OTUb65 (1)
OTUb464 (1)
AY921922, AKYH548
OTUb374 (1)
OTUb614 (1)
AB072735, Gemmatimonas aurantiaca
Gemmatimonadetes
OTUb246 (1)
EU804764, 6C232724
FJ745242, SHWN_night2_16S_453
JX537830, 63-38
OTUb412 (1,1,1)
CP000825, Prochlorococcus marinus
DQ070784, 9NSWBact_8 16S
Cyanobacteria
U00096, Escherichia coli
CP000926, Pseudomonas putida
0.10

Fig. S3D

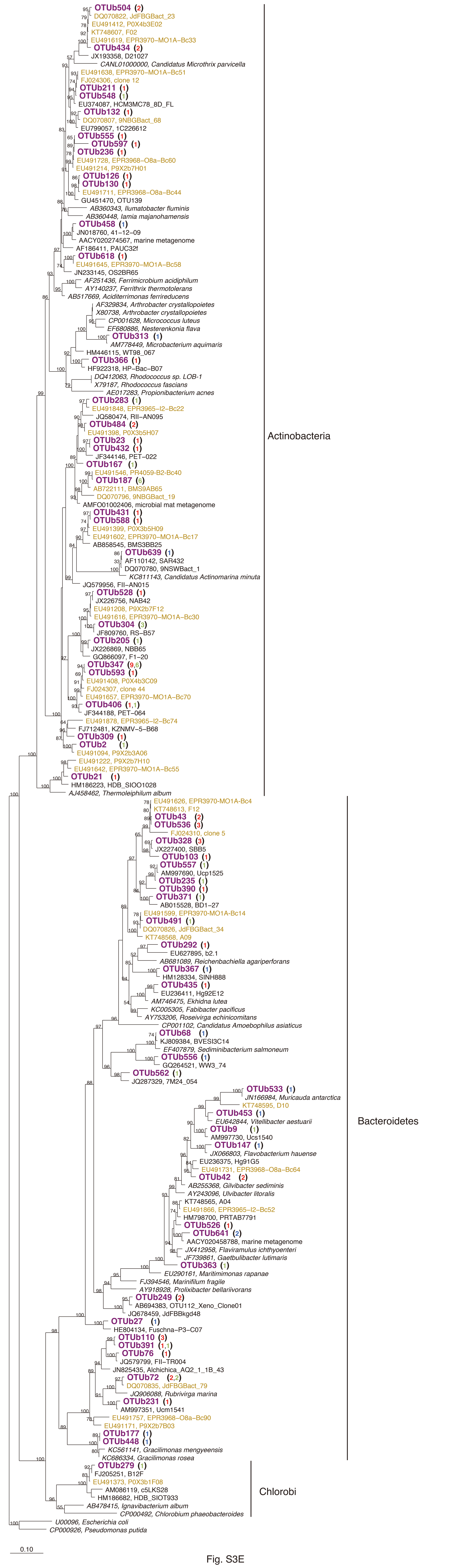

OTUb504 (2)
DQ070822, JdFBGBact_23
EU491412, P0X4b3E02
KT748607, F02
EU491619, EPR3970−MO1A−Bc33
OTUb434 (2)
JX193358, D21027
CANL01000000, Candidatus Microthrix parvicella
EU491638, EPR3970−MO1A−Bc51
FJ024306, clone 12
OTUb211 (1)
OTUb548 (1)
EU374087, HCM3MC78_8D_FL
OTUb132 (1)
DQ070807, 9NBGBact_68
EU799057, 1C226612
OTUb555 (1)
OTUb597 (1)
OTUb236 (1)
EU491728, EPR3968−O8a−Bc60
EU491214, P9X2b7H01
OTUb126 (1)
OTUb130 (1)
EU491711, EPR3968−O8a−Bc44
GU451470, OTU139
AB360343, Ilumatobacter fluminis
AB360448, Iamia majanohamensis
OTUb458 (1)
JN018760, 41−12−09
AACY020274567, marine metagenome
AF186411, PAUC32f
OTUb618 (1)
EU491645, EPR3970−MO1A−Bc58
JN233145, OS2BR65
AF251436, Ferrimicrobium acidiphilum
AY140237, Ferrithrix thermotolerans
AB517669, Aciditerrimonas ferrireducens
AF329834, Arthrobacter crystallopoietes
X80738, Arthrobacter crystallopoietes
CP001628, Micrococcus luteus
EF680886, Nesterenkonia flava
OTUb313 (1)
AM778449, Microbacterium aquimaris
HM446115, WT98_067
OTUb366 (1)
HF922318, HP−Bac−B07
DQ412063, Rhodococcus sp. LOB-1
X79187, Rhodococcus fascians
AE017283, Propionibacterium acnes
OTUb283 (1)
EU491848, EPR3965−I2−Bc22
JQ580474, RII−AN095
OTUb484 (2)
EU491398, P0X3b5H07
OTUb23 (1)
OTUb432 (1)
JF344146, PET−022
OTUb167 (1)
EU491546, PR4059-B2-Bc40
OTUb187 (6)
AB722111, BMS9AB65
DQ070796, 9NBGBact_19
AMFO01002406, microbial mat metagenome
OTUb431 (1)
OTUb588 (1)
EU491399, P0X3b5H09
EU491602, EPR3970−MO1A−Bc17
AB858545, BMS3BB25
OTUb639 (1)
AF110142, SAR432
DQ070780, 9NSWBact_1
KC811143, Candidatus Actinomarina minuta
JQ579956, FII−AN015
OTUb528 (1)
JX226756, NAB42
EU491208, P9X2b7F12
EU491616, EPR3970−MO1A−Bc30
OTUb304 (3)
JF809760, RS−B57
OTUb205 (1)
JX226869, NBB65
GQ866097, F1−20
OTUb347 (9,6)
OTUb593 (1)
EU491408, P0X4b3C09
FJ024307, clone 44
EU491657, EPR3970−MO1A−Bc70
OTUb406 (1,1)
JF344188, PET−064
EU491878, EPR3965−I2−Bc74
FJ712481, KZNMV−5−B68
OTUb309 (1)
OTUb2 (1)
EU491094, P9X2b3A06
EU491222, P9X2b7H10
EU491642, EPR3970−MO1A−Bc55
OTUb21 (1)
HM186223, HDB_SIOO1028
AJ458462, Thermoleiphilum album
Actinobacteria
EU491626, EPR3970-MO1A-Bc4
KT748613, F12
OTUb43 (2)
OTUb536 (3)
FJ024310, clone 5
OTUb328 (3)
JX227400, SBB5
OTUb103 (1)
OTUb557 (1)
AM997690, Ucp1525
OTUb235 (1)
OTUb390 (1)
OTUb371 (1)
AB015528, BD1−27
EU491599, EPR3970-MO1A-Bc14
OTUb491 (1)
DQ070826, JdFBGBact_34
KT748568, A09
OTUb292 (1)
EU627895, b2.1
AB681089, Reichenbachiella agariperforans
OTUb367 (1)
HM128334, SINH888
OTUb435 (1)
EU236411, Hg92E12
AM746475, Ekhidna lutea
KC005305, Fabibacter pacificus
AY753206, Roseivirga echinicomitans
CP001102, Candidatus Amoebophilus asiaticus
OTUb68 (1)
KJ809384, BVESI3C14
EF407879, Sediminibacterium salmoneum
OTUb556 (1)
GQ264521, WW3_74
OTUb562 (1)
JQ287329, 7M24_054
OTUb533 (1)
JN166984, Muricauda antarctica
KT748595, D10
OTUb453 (1)
EU642844, Vitellibacter aestuarii
OTUb9 (1)
AM997730, Ucs1540
OTUb147 (1)
JX066803, Flavobacterium hauense
EU236375, Hg91G5
EU491731, EPR3968−O8a−Bc64
OTUb42 (2)
AB255368, Gilvibacter sediminis
AY243096, Ulvibacter litoralis
KT748565, A04
EU491866, EPR3965−I2−Bc52
HM798700, PRTAB7791
OTUb526 (1)
OTUb641 (2)
AACY020458788, marine metagenome
JX412958, Flaviramulus ichthyoenteri
JF739861, Gaetbulibacter lutimaris
OTUb363 (1)
EU290161, Maritimimonas rapanae
FJ394546, Marinifilum fragile
AY918928, Prolixibacter bellariivorans
OTUb249 (2)
AB694383, OTU112_Xeno_Clone01
JQ678459, JdFBBkgd48
OTUb27 (1)
HE804134, Fuschna−P3−C07
OTUb110 (3)
OTUb391 (1,1)
OTUb76 (1)
JQ579799, FII−TR004
JN825435, Alchichica_AQ2_1_1B_43
OTUb72 (2,2)
DQ070835, JdFBGBact_79
JQ906088, Rubrivirga marina
OTUb231 (1)
AM997351, Ucm1541
EU491757, EPR3968−O8a−Bc90
EU491171, P9X2b7B03
OTUb177 (1)
OTUb448 (1)
KC561141, Gracilimonas mengyeensis
KC686334, Gracilimonas rosea
Bacteroidetes
OTUb279 (1)
FJ205251, B12F
EU491373, P0X3b1F08
AM086119, c5LKS28
HM186682, HDB_SIOT933
AB478415, Ignavibacterium album
CP000492, Chlorobium phaeobacteroides
Chlorobi
U00096, Escherichia coli
CP000926, Pseudomonas putida
0.10

Fig. S3E

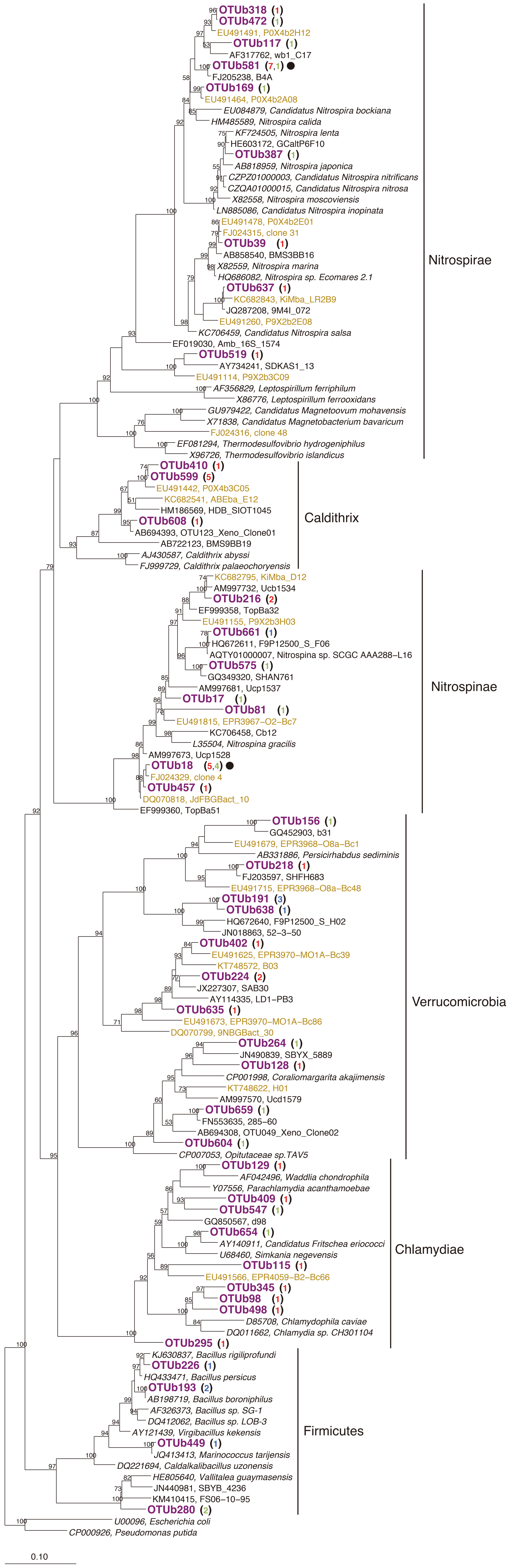
OTUb318 (1)
OTUb472 (1)
EU491491, P0X4b2H12
OTUb117 (1)
AF317762, wb1_C17
OTUb581 (7,1)
FJ205238, B4A
OTUb169 (1)
EU491464, P0X4b2A08
EU084879, Candidatus Nitrospira bockiana
HM485589, Nitrospira calida
KF724505, Nitrospira lenta
HE603172, GCaltP6F10
OTUb387 (1)
AB818959, Nitrospira japonica
CZPZ01000003, Candidatus Nitrospira nitrificans
CZQA01000015, Candidatus Nitrospira nitrosa
X82558, Nitrospira moscoviensis
LN885086, Candidatus Nitrospira inopinata
EU491478, P0X4b2E01
FJ024315, clone 31
OTUb39 (1)
AB858540, BMS3BB16
X82559, Nitrospira marina
HQ686082, Nitrospira sp. Ecomares 2.1
OTUb637 (1)
KC682843, KiMba_LR2B9
JQ287208, 9M4I_072
EU491260, P9X2b2E08
KC706459, Candidatus Nitrospira salsa
EF019030, Amb_16S_1574
OTUb519 (1)
AY734241, SDKAS1_13
EU491114, P9X2b3C09
AF356829, Leptospirillum ferriphilum
X86776, Leptospirillum ferrooxidans
GU979422, Candidatus Magnetoovum mohavensis
X71838, Candidatus Magnetobacterium bavaricum
FJ024316, clone 48
EF081294, Thermodesulfovibrio hydrogeniphilus
X96726, Thermodesulfovibrio islandicus
Nitrospirae
OTUb410 (1)
OTUb599 (5)
EU491442, P0X4b3C05
KC682541, ABEba_E12
HM186569, HDB_SIOT1045
OTUb608 (1)
AB694393, OTU123_Xeno_Clone01
AB722123, BMS9BB19
AJ430587, Caldithrix abyssi
FJ999729, Caldithrix palaeochoryensis
Caldithrix
KC682795, KiMba_D12
AM997732, Ucb1534
OTUb216 (2)
EF999358, TopBa32
EU491155, P9X2b3H03
OTUb661 (1)
HQ672611, F9P12500_S_F06
AQTY01000007, Nitrospina sp. SCGC AAA288−L16
OTUb575 (1)
GQ349320, SHAN761
AM997681, Ucp1537
OTUb17 (1)
OTUb81 (1)
EU491815, EPR3967−O2−Bc7
KC706458, Cb12
L35504, Nitrospina gracilis
AM997673, Ucp1528
OTUb18 (5,4)
FJ024329, clone 4
OTUb457 (1)
DQ070818, JdFBGBact_10
EF999360, TopBa51
Nitrospinae
OTUb156 (1)
GQ452903, b31
EU491679, EPR3968−O8a−Bc1
AB331886, Persicirhabdus sediminis
OTUb218 (1)
FJ203597, SHFH683
EU491715, EPR3968−O8a−Bc48
OTUb191 (3)
OTUb638 (1)
HQ672640, F9P12500_S_H02
JN018863, 52−3−50
OTUb402 (1)
EU491625, EPR3970−MO1A−Bc39
KT748572, B03
OTUb224 (2)
JX227307, SAB30
AY114335, LD1−PB3
OTUb635 (1)
EU491673, EPR3970−MO1A−Bc86
DQ070799, 9NBGBact_30
OTUb264 (1)
JN490839, SBYX_5889
OTUb128 (1)
CP001998, Coraliomargarita akajimensis
KT748622, H01
AM997570, Ucd1579
OTUb659 (1)
FN553635, 285−60
AB694308, OTU049_Xeno_Clone02
OTUb604 (1)
CP007053, Opitutaceae sp.TAV5
Verrucomicrobia
OTUb129 (1)
AF042496, Waddlia chondrophila
Y07556, Parachlamydia acanthamoebae
OTUb409 (1)
OTUb547 (1)
GQ850567, d98
OTUb654 (1)
AY140911, Candidatus Fritschea eriococci
U68460, Simkania negevensis
OTUb115 (1)
EU491566, EPR4059−B2−Bc66
OTUb345 (1)
OTUb98 (1)
OTUb498 (1)
D85708, Chlamydophila caviae
DQ011662, Chlamydia sp. CH301104
OTUb295 (1)
Chlamydiae
KJ630837, Bacillus rigiliprofundi
OTUb226 (1)
HQ433471, Bacillus persicus
OTUb193 (2)
AB198719, Bacillus boroniphilus
AF326373, Bacillus sp. SG-1
DQ412062, Bacillus sp. LOB-3
AY121439, Virgibacillus kekensis
OTUb449 (1)
JQ413413, Marinococcus tarijensis
DQ221694, Caldalkalibacillus uzonensis
HE805640, Vallitalea guaymasensis
JN440981, SBYB_4236
KM410415, FS06−10−95
OTUb280 (2)
Firmicutes
U00096, Escherichia coli
CP000926, Pseudomonas putida
0.10

Fig. S3F

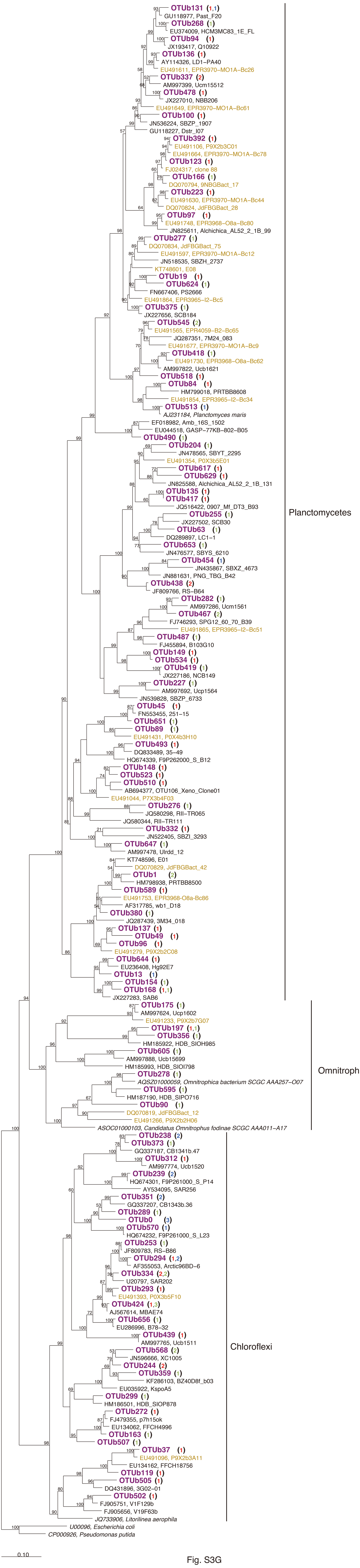
OTUb131 (1,1)
GU118977, Past_F20
OTUb268 (1)
EU374009, HCM3MC83_1E_FL
OTUb94 (1)
JX193417, Q10922
OTUb136 (1)
AY114326, LD1-PA40
EU491611, EPR3970-MO1A-Bc26
OTUb337 (2)
AM997399, Ucm15512
OTUb478 (1)
JX227010, NBB206
EU491649, EPR3970-MO1A-Bc61
OTUb100 (1)
JN536224, SBZP_1907
GU118227, Dstr_I07
OTUb392 (1)
EU491106, P9X2b3C01
EU491664, EPR3970-MO1A-Bc78
OTUb123 (1)
FJ024317, clone 88
OTUb166 (1)
DQ070794, 9NBGBact_17
OTUb223 (1)
EU491630, EPR3970-MO1A-Bc44
DQ070824, JdFBGBact_28
OTUb97 (1)
EU491748, EPR3968-O8a-Bc80
JN825611, Alchichica_AL52_2_1B_99
OTUb277 (1)
DQ070834, JdFBGBact_75
EU491597, EPR3970-MO1A-Bc12
JN518535, SBZH_2737
KT748601, E08
OTUb19 (1)
OTUb624 (1)
FN667406, PS2666
EU491864, EPR3965-I2-Bc5
OTUb375 (1)
JX227656, SCB184
OTUb545 (2)
EU491565, EPR4059-B2-Bc65
JQ287351, 7M24_083
EU491677, EPR3970-MO1A-Bc9
OTUb418 (1)
EU491730, EPR3968-O8a-Bc62
AM997822, Ucb1621
OTUb518 (1)
OTUb84 (1)
HM799018, PRTBB8608
EU491854, EPR3965-I2-Bc34
OTUb513 (1)
AJ231184, Planctomyces maris
EF018982, Amb_16S_1502
EU044518, GASP-77KB-802-B05
OTUb490 (1)
OTUb204 (1)
JN478565, SBYT_2295
EU491354, P0X3b5E01
OTUb617 (1)
OTUb629 (1)
JN825588, Alchichica_AL52_2_1B_131
OTUb135 (1)
OTUb417 (1)
JQ516422, 0907_Mf_DT3_B93
OTUb255 (1)
JX227502, SCB30
OTUb63 (1)
DQ289897, LC1-1
OTUb653 (1)
JN476577, SBYS_6210
OTUb454 (1)
JN435867, SBXZ_4673
JN881631, PNG_TBG_B42
OTUb438 (2)
JF809766, RS-B64
OTUb282 (1)
AM997286, Ucm1561
OTUb467 (2)
FJ746293, SPG12_60_70_B39
EU491865, EPR3965-I2-Bc51
OTUb487 (1)
FJ455894, B103G10
OTUb149 (1)
OTUb534 (1)
OTUb419 (1)
JX227186, NCB149
OTUb227 (1)
AM997692, Ucp1564
JN539828, SBZP_6733
OTUb45 (1)
FN553455, 251-15
OTUb651 (1)
OTUb89 (1)
EU491431, P0X4b3H10
OTUb493 (1)
DQ833489, 35-49
HQ674339, F9P262000_S_B12
OTUb148 (1)
OTUb523 (1)
OTUb510 (1)
AB694377, OTU106_Xeno_Clone01
EU491044, P7X3b4F03
OTUb276 (1)
JQ580298, RII-TR065
JQ580344, RII-TR111
OTUb332 (1)
JN522405, SBZI_3293
OTUb647 (1)
AM997478, Ulrdd_12
KT748596, E01
DQ070829, JdFBGBact_42
OTUb1 (2)
HM798938, PRTBB8500
OTUb589 (1)
EU491753, EPR3968-O8a-Bc86
AF317785, wb1_D18
OTUb380 (1)
JQ287439, 3M34_018
OTUb137 (1)
OTUb49 (1)
OTUb96 (1)
EU491279, P9X2b2C08
OTUb644 (1)
EU236408, Hg92E7
OTUb13 (1)
OTUb154 (1)
OTUb168 (1,1)
JX227283, SAB6
Planctomycetes
OTUb175 (1)
AM997624, Ucp1602
EU491233, P9X2b7G07
OTUb197 (1,1)
OTUb356 (1)
HM185922, HDB_SIOH985
OTUb605 (1)
AM997888, Ucb15699
HM185993, HDB_SIOI798
OTUb278 (1)
AQSZ01000059, Omnitrophica bacterium SCGC AAA257-O07
OTUb595 (1)
HM187190, HDB_SIPO716
OTUb90 (1)
DQ070819, JdFBGBact_12
EU491266, P9X2b2H06
ASOC01000103, Candidatus Omnitrophus fodinae SCGC AAA011-A17
Omnitrophica (OP3)
OTUb238 (2)
OTUb373 (1)
GQ337187, CB1341b.47
OTUb312 (1)
AM997774, Ucb1520
OTUb239 (2)
HQ674301, F9P261000_S_P14
AY534095, SAR256
OTUb351 (2)
GQ337207, CB1343b.36
OTUb289 (1)
OTUb0 (3)
OTUb570 (1)
HQ674232, F9P261000_S_L23
OTUb253 (1)
JF809783, RS-B86
OTUb294 (1,2)
AF355053, Arctic96BD-6
OTUb334 (2,2)
U20797, SAR202
OTUb293 (1)
EU491393, P0X3b5F10
OTUb424 (1,3)
AJ567614, MBAE74
OTUb656 (1)
EU286996, B78-32
OTUb439 (1)
AM997765, Ucb1511
OTUb568 (2)
JN596666, XC1005
OTUb244 (2)
OTUb359 (1)
KF286103, BZ40D8f_b03
EU035922, KspoA5
OTUb299 (1)
HM186501, HDB_SIOP878
OTUb272 (1)
FJ479355, p7h15ok
EU134062, FFCH4996
OTUb163 (1)
OTUb507 (1)
OTUb37 (1)
EU491096, P9X2b3A11
EU134162, FFCH18756
OTUb119 (1)
OTUb505 (1)
DQ431896, 3G02-01
OTUb502 (1)
FJ905751, V1F129b
FJ905656, V19F63b
JQ733906, Litorilinea aerophila
Chloroflexi
U00096, Escherichia coli
CP000926, Pseudomonas putida
0.10

Fig. S3G

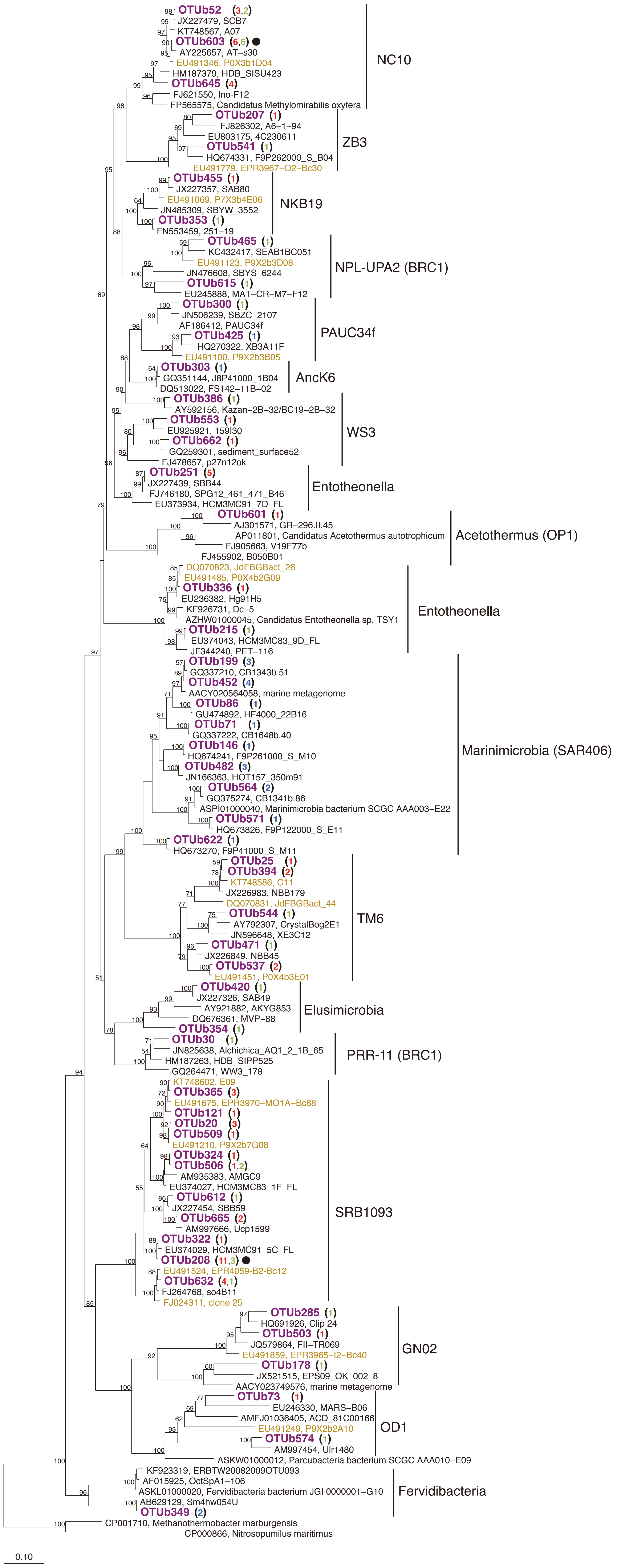
OTUb52 (3,2)
JX227479, SCB7
KT748567, A07
OTUb603 (6,6)
AY225657, AT-s30
EU491346, P0X3b1D04
HM187379, HDB_SISU423
OTUb645 (4)
FJ621550, Ino-F12
FP565575, Candidatus Methylomirabilis oxyfera
NC10
OTUb207 (1)
FJ826302, A6-1-94
EU803175, 4C230611
OTUb541 (1)
HQ674331, F9P262000_S_B04
EU491779, EPR3967-O2-Bc30
ZB3
OTUb455 (1)
JX227357, SAB80
EU491069, P7X3b4E06
JN485309, SBYW_3552
OTUb353 (1)
FN553459, 251-19
NKB19
OTUb465 (1)
KC432417, SEAB1BC051
EU491123, P9X2b3D08
JN476608, SBYS_6244
OTUb615 (1)
EU245888, MAT-CR-M7-F12
NPL-UPA2 (BRC1)
OTUb300 (1)
JN506239, SBZC_2107
AF186412, PAUC34f
OTUb425 (1)
HQ270322, XB3A11F
EU491100, P9X2b3B05
PAUC34f
OTUb303 (1)
GQ351144, J8P41000_1B04
DQ513022, FS142-11B-02
AncK6
OTUb386 (1)
AY592156, Kazan-2B-32/BC19-2B-32
OTUb553 (1)
EU925921, 159I30
OTUb662 (1)
GQ259301, sediment_surface52
FJ478657, p27n12ok
WS3
OTUb251 (5)
JX227439, SBB44
FJ746180, SPG12_461_471_B46
EU373934, HCM3MC91_7D_FL
Entotheonella
OTUb601 (1)
AJ301571, GR-296.II.45
AP011801, Candidatus Acetothermus autotrophicum
FJ905663, V19F77b
FJ455902, B050B01
Acetothermus (OP1)
DQ070823, JdFBGBact_26
EU491485, P0X4b2G09
OTUb336 (1)
EU236382, Hg91H5
KF926731, Dc-5
AZHW01000045, Candidatus Entotheonella sp. TSY1
OTUb215 (1)
EU374043, HCM3MC83_9D_FL
JF344240, PET-116
Entotheonella
OTUb199 (3)
GQ337210, CB1343b.51
OTUb452 (4)
AACY020564058, marine metagenome
OTUb86 (1)
GU474892, HF4000_22B16
OTUb71 (1)
GQ337222, CB1648b.40
OTUb146 (1)
HQ674241, F9P261000_S_M10
OTUb482 (3)
JN166363, HOT157_350m91
OTUb564 (2)
GQ375274, CB1341b.86
ASPI01000040, Marinimicrobia bacterium SCGC AAA003-E22
OTUb571 (1)
HQ673826, F9P122000_S_E11
OTUb622 (1)
HQ673270, F9P41000_S_M11
Marinimicrobia (SAR406)
OTUb25 (1)
OTUb394 (2)
KT748586, C11
JX226983, NBB179
DQ070831, JdFBGBact_44
OTUb544 (1)
AY792307, CrystalBog2E1
JN596648, XE3C12
OTUb471 (1)
JX226849, NBB45
OTUb537 (2)
EU491451, P0X4b3E01
TM6
OTUb420 (1)
JX227326, SAB49
AY921882, AKYG853
DQ676361, MVP-88
OTUb354 (1)
Elusimicrobia
OTUb30 (1)
JN825638, Alchichica_AQ1_2_1B_65
HM187263, HDB_SIPP525
GQ264471, WW3_178
PRR-11 (BRC1)
KT748602, E09
OTUb365 (3)
EU491675, EPR3970-MO1A-Bc88
OTUb121 (1)
OTUb20 (3)
OTUb509 (1)
EU491210, P9X2b7G08
OTUb324 (1)
OTUb506 (1,2)
AM935383, AMGC9
EU374027, HCM3MC83_1F_FL
OTUb612 (1)
JX227454, SBB59
OTUb665 (2)
AM997666, Ucp1599
OTUb322 (1)
EU374029, HCM3MC91_5C_FL
OTUb208 (11,3)
EU491524, EPR4059-B2-Bc12
OTUb632 (4,1)
FJ264768, so4B11
FJ024311, clone 25
SRB1093
OTUb285 (1)
HQ691926, Clip 24
OTUb503 (1)
JQ579864, FII-TR069
EU491859, EPR3965-I2-Bc40
OTUb178 (1)
JX521515, EPS09_OK_002_8
AACY023749576, marine metagenome
GN02
OTUb73 (1)
EU246330, MARS-B06
AMFJ01036405, ACD_81C00166
EU491249, P9X2b2A10
OTUb574 (1)
AM997454, Ulr1480
ASKW01000012, Parcubacteria bacterium SCGC AAA010-E09
OD1
KF923319, ERBTW20082009OTU093
AF015925, OctSpA1-106
ASKL01000020, Fervidibacteria bacterium JGI 0000001-G10
AB629129, Sm4hw054U
OTUb349 (2)
Fervidibacteria
CP001710, Methanothermobacter marburgensis
CP000866, Nitrosopumilus maritimus
0.10

Fig. S3H

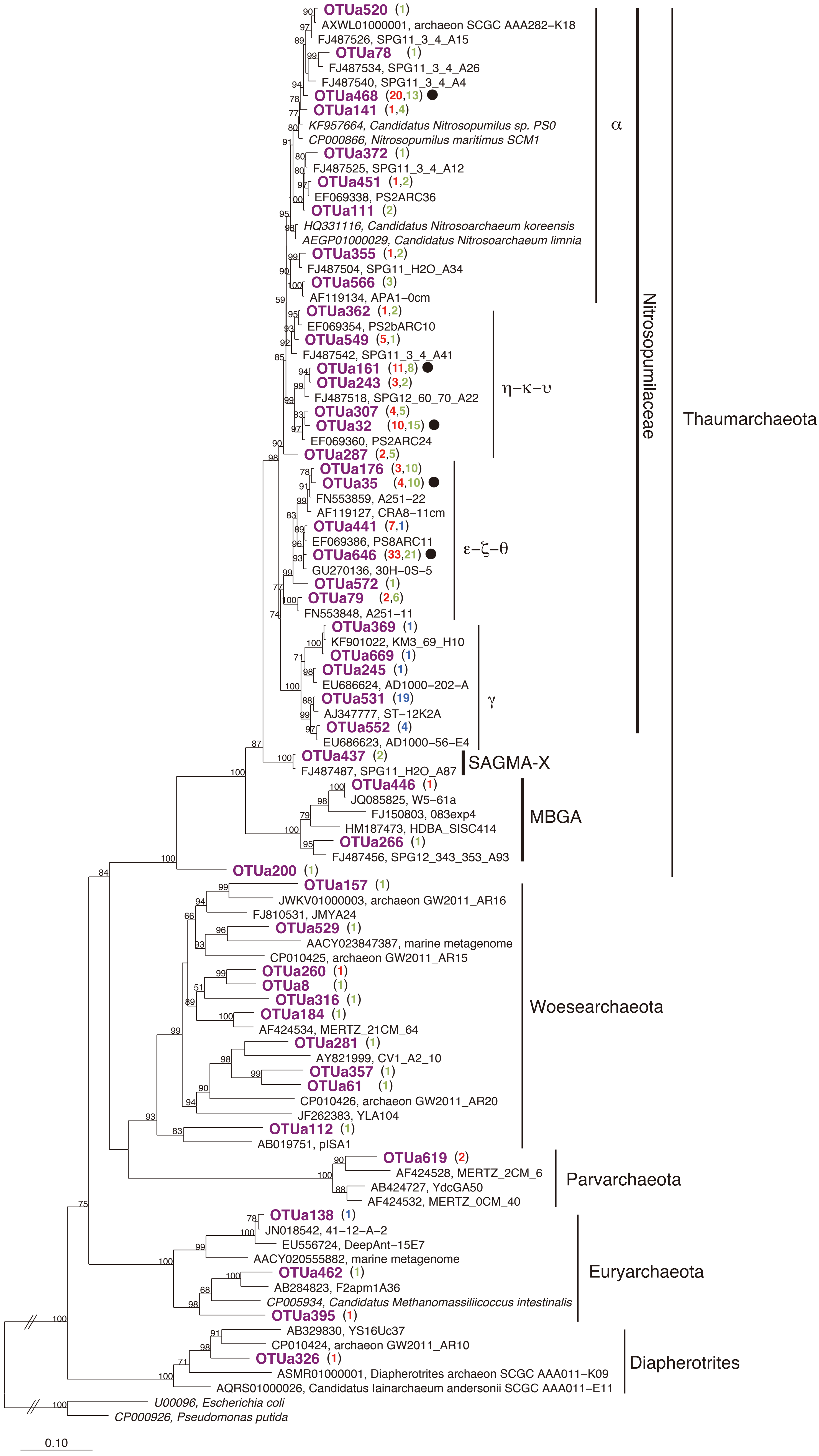
OTUa520 (1)
AXWL01000001, archaeon SCGC AAA282−K18
FJ487526, SPG11_3_4_A15
OTUa78 (1)
FJ487534, SPG11_3_4_A26
FJ487540, SPG11_3_4_A4
OTUa468 (20,13)
OTUa141 (1,4)
KF957664, Candidatus Nitrosopumilus sp. PS0
CP000866, Nitrosopumilus maritimus SCM1
OTUa372 (1)
FJ487525, SPG11_3_4_A12
OTUa451 (1,2)
EF069338, PS2ARC36
OTUa111 (2)
HQ331116, Candidatus Nitrosoarchaeum koreensis
AEGP01000029, Candidatus Nitrosoarchaeum limnia
OTUa355 (1,2)
FJ487504, SPG11_H2O_A34
OTUa566 (3)
AF119134, APA1−0cm
OTUa362 (1,2)
EF069354, PS2bARC10
OTUa549 (5,1)
FJ487542, SPG11_3_4_A41
OTUa161 (11,8)
OTUa243 (3,2)
FJ487518, SPG12_60_70_A22
OTUa307 (4,5)
OTUa32 (10,15)
EF069360, PS2ARC24
OTUa287 (2,5)
OTUa176 (3,10)
OTUa35 (4,10)
FN553859, A251−22
AF119127, CRA8−11cm
OTUa441 (7,1)
EF069386, PS8ARC11
OTUa646 (33,21)
GU270136, 30H−0S−5
OTUa572 (1)
OTUa79 (2,6)
FN553848, A251−11
OTUa369 (1)
KF901022, KM3_69_H10
OTUa669 (1)
OTUa245 (1)
EU686624, AD1000−202−A
OTUa531 (19)
AJ347777, ST−12K2A
OTUa552 (4)
EU686623, AD1000−56−E4
OTUa437 (2)
FJ487487, SPG11_H2O_A87
OTUa446 (1)
JQ085825, W5−61a
FJ150803, 083exp4
HM187473, HDBA_SISC414
OTUa266 (1)
FJ487456, SPG12_343_353_A93
OTUa200 (1)
OTUa157 (1)
JWKV01000003, archaeon GW2011_AR16
FJ810531, JMYA24
OTUa529 (1)
AACY023847387, marine metagenome
CP010425, archaeon GW2011_AR15
OTUa260 (1)
OTUa8 (1)
OTUa316 (1)
OTUa184 (1)
AF424534, MERTZ_21CM_64
OTUa281 (1)
AY821999, CV1_A2_10
OTUa357 (1)
OTUa61 (1)
CP010426, archaeon GW2011_AR20
JF262383, YLA104
OTUa112 (1)
AB019751, pISA1
OTUa619 (2)
AF424528, MERTZ_2CM_6
AB424727, YdcGA50
AF424532, MERTZ_0CM_40
OTUa138 (1)
JN018542, 41−12−A−2
EU556724, DeepAnt−15E7
AACY020555882, marine metagenome
OTUa462 (1)
AB284823, F2apm1A36
CP005934, Candidatus Methanomassiliicoccus intestinalis
OTUa395 (1)
AB329830, YS16Uc37
CP010424, archaeon GW2011_AR10
OTUa326 (1)
ASMR01000001, Diapherotrites archaeon SCGC AAA011−K09
AQRS01000026, Candidatus Iainarchaeum andersonii SCGC AAA011−E11
U00096, Escherichia coli
CP000926, Pseudomonas putida
0.10
α
η−κ−υ
ε−ζ−θ
γ
SAGMA-X
MBGA
Nitrosopumilaceae
Thaumarchaeota
Woesearchaeota
Parvarchaeota
Euryarchaeota
Diapherotrites

Fig. S3I
