# Supplementary material for: Archaeal and bacterial communities in deep-sea hydrogenetic ferromanganese crusts on old seamounts of the northwestern Pacific
Source: PLoS One. 2017 Feb 24;12(2):e0173071. doi: 10.1371/journal.pone.0173071 (PMC5325594; doi:10.1371/journal.pone.0173071)
Supplement: S5 Fig — (A) Among habitat types, (B) among regions for the crust samples, and (C) among water depths for the crust samples of the Takuyo-Daigo Seamount. Numbers in circles and in parentheses are the numbers of OTUs. The names and taxonomic affiliations of the common OTUs among them are indicated. The last two letters of the sample name indicate the approximate sampling depth in hundred meters. Numbers in circles and in parentheses are the numbers of OTUs. The last two letters of the sample name indicate the approximate sampling depth in hundred meters. (PDF) [file pone.0173071.s005.pdf]

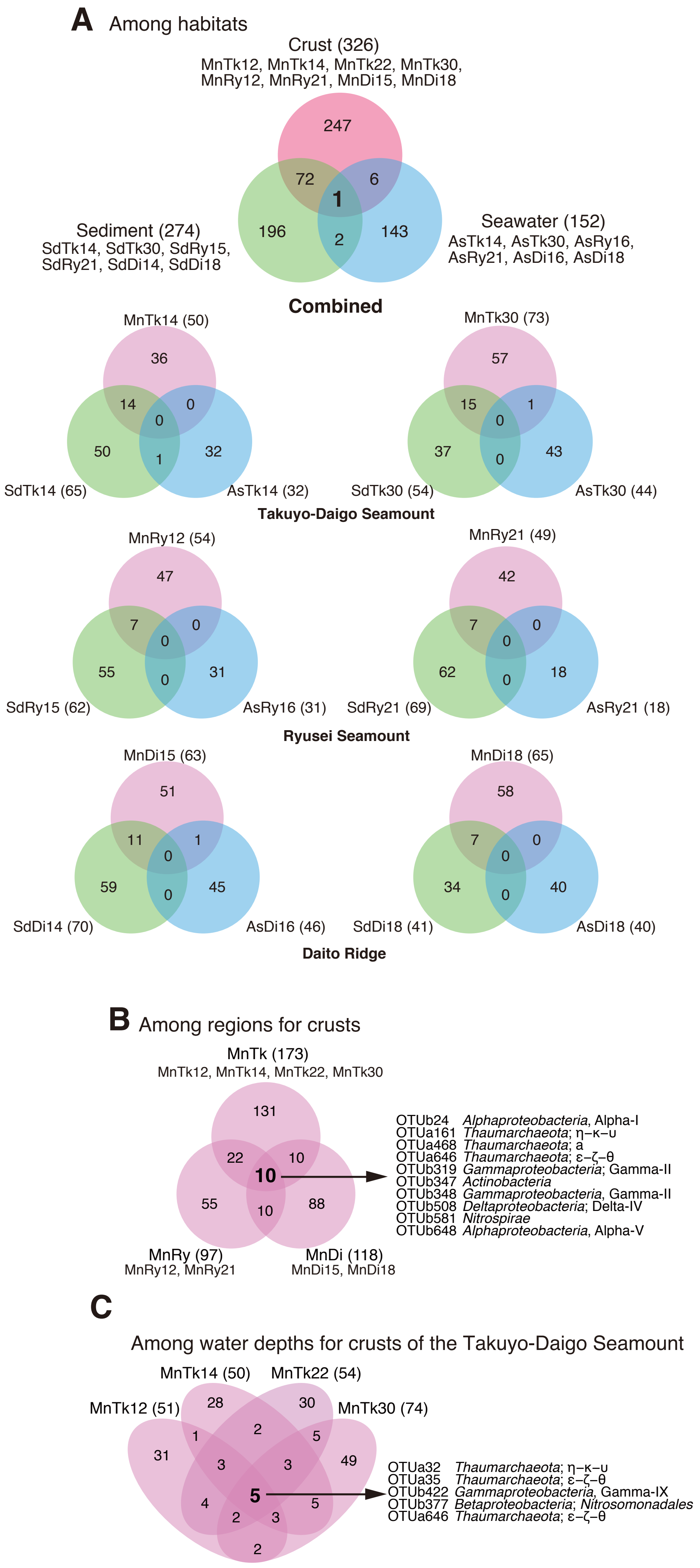
A Among habitats
Crust (326)
MnTk12, MnTk14, MnTk22, MnTk30,
MnRy12, MnRy21, MnDi15, MnDi18
247
72
6
1
196
2
143
Sediment (274)
SdTk14, SdTk30, SdRy15,
SdRy21, SdDi14, SdDi18
Seawater (152)
AsTk14, AsTk30, AsRy16,
AsRy21, AsDi16, AsDi18
Combined
MnTk14 (50)
36
14
0
0
50
1
32
SdTk14 (65)
AsTk14 (32)
MnTk30 (73)
57
15
1
0
37
0
43
SdTk30 (54)
AsTk30 (44)
Takuyo-Daigo Seamount
MnRy12 (54)
47
7
0
0
55
0
31
SdRy15 (62)
AsRy16 (31)
MnRy21 (49)
42
7
0
0
62
0
18
SdRy21 (69)
AsRy21 (18)
Ryusei Seamount
MnDi15 (63)
51
11
1
0
59
0
45
SdDi14 (70)
AsDi16 (46)
MnDi18 (65)
58
7
0
0
34
0
40
SdDi18 (41)
AsDi18 (40)
Daito Ridge
B Among regions for crusts
MnTk (173)
MnTk12, MnTk14, MnTk22, MnTk30
131
22
10
10
55
10
88
MnRy (97)
MnRy12, MnRy21
MnDi (118)
MnDi15, MnDi18
OTUb24 *Alphaproteobacteria*, Alpha-I
OTUa161 *Thaumarchaeota*; η–κ–υ
OTUa468 *Thaumarchaeota*; a
OTUa646 *Thaumarchaeota*; ε–ζ–θ
OTUb319 *Gammaproteobacteria*; Gamma-II
OTUb347 *Actinobacteria*
OTUb348 *Gammaproteobacteria*, Gamma-II
OTUb508 *Deltaproteobacteria*; Delta-IV
OTUb581 *Nitrospirae*
OTUb648 *Alphaproteobacteria*, Alpha-V
C
Among water depths for crusts of the Takuyo-Daigo Seamount
MnTk14 (50)
MnTk22 (54)
MnTk12 (51)
MnTk30 (74)
28
30
1
2
5
31
3
3
49
5
4
2
3
5
2
OTUa32 *Thaumarchaeota*; η–κ–υ
OTUa35 *Thaumarchaeota*; ε–ζ–θ
OTUb422 *Gammaproteobacteria*, Gamma-IX
OTUb377 *Betaproteobacteria*; *Nitrosomonadales*
OTUa646 *Thaumarchaeota*; ε–ζ–θ

Fig. S5
